# Acid-enhanced RAFT depolymerization of polymethacrylates

**DOI:** 10.1039/d6sc05503f

**Published:** 2026-09-24

**Authors:** Zuzanna Kantor, Maria-Nefeli Antonopoulou, Athénaïs Pascal-Touraine, Asja A. Kroeger, Nghia P. Truong, Glen R. Jones, Michelle L. Coote, Athina Anastasaki

**Affiliations:** a Laboratory for Sustainable Polymers, Department of Materials, ETH Zürich Vladimir-Prelog-Weg 5 8093 Zürich Switzerland m-nef@windowslive.com athina.anastasaki@mat.ethz.ch; b Institute for Nanoscale Science and Technology, College of Science and Engineering, Flinders University South Australia 5042 Australia

## Abstract

While acidic additives have recently been shown to significantly enhance vinyl polymerizations, their effects on the reverse depropagation pathway remain completely unexplored. Herein, acid-enhanced RAFT depolymerization is introduced as a means to effectively replace thermal radical initiators with abundant acids, while also enhancing the depolymerization rate and efficiency. In particular, the addition of a simple Brønsted acid, such as acetic acid, provides up to a 7.6-fold increase in the reaction rate and more than doubles the final conversion at elevated concentrations, increasing the yield from 34% (without an acidic additive) to 75%. This accelerating effect was attributed to the acid directly lowering the activation barrier for the depropagation step, as supported by complementary density functional theory calculations. In addition, the enhanced conversion was linked to the greater end-group preservation in the presence of acid, as indicated by the retention of the UV signal during size exclusion chromatography measurements. Overall, this strategy is highly robust and versatile, operating across varied Brønsted acid strengths, such as trifluoroacetic acid and sustainable citric acid, and extending to Lewis acid catalysis at ultra-low loadings. The introduced methodology simplifies current RAFT depolymerization protocols, while revealing unique mechanistic insights concerning the role of acid during the chemical recycling of polymethacrylates.

## Abstract 2

Depolymerization of RAFT-synthesized polymers in the presence of acid increases reaction rates up to 7.6-fold and increases monomer yield by preserving end groups and lowering depropagation barriers.

## Graphical abstract

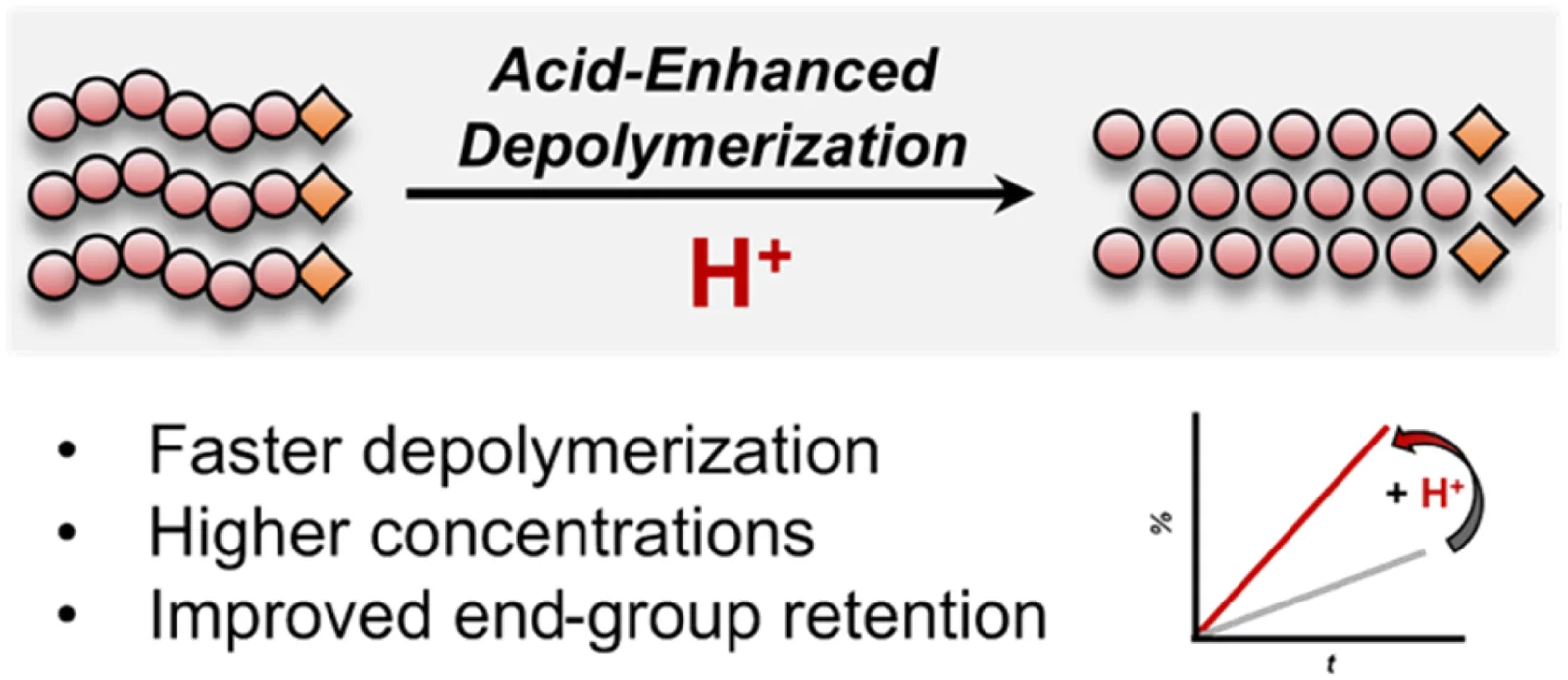

## Introduction

Reversible deactivation radical polymerization (RDRP), also known as controlled radical polymerization (CRP), is a powerful tool for material synthesis, enabling precise control over molecular weight, end-group fidelity, dispersity, and macromolecular architecture.^1,2^ Amongst these advantageous features, the retention of high end-group fidelity is of paramount importance not only for the effective synthesis of block copolymers,^3,4^ but also, more recently, to trigger the low temperature depolymerization of polymethacrylates.^5,6^ By employing lower temperatures than traditional pyrolysis, early reports leveraged heat exclusively as an external stimulus to initially activate the chain ends and subsequently trigger the depropagation of the polymer chains under thermodynamically favorable conditions.^7^ While the chemical recycling of polymers synthesized by atom transfer radical polymerization (ATRP) requires a metal catalyst, typically copper or iron, to achieve an efficient depolymerization at 170 °C,^8,9^ the depolymerization of reversible addition–fragmentation chain-transfer (RAFT)-synthesized materials typically proceeds at relatively lower temperatures (*i.e.*, 120 °C). To this end, several research groups have demonstrated effective depolymerizations under a range of reaction conditions, targeting high percentages of monomer recovery and fast depolymerization rates.^10–14^

More recently, alternative external stimuli have also been explored with the aim to expand the depolymerization scope, offer additional benefits such as temporal control, and investigate intriguing mechanistic pathways.^15^ Perhaps not surprisingly, light was first examined as an efficient external stimulus to enable a lower temperature ATRP depolymerization while inducing temporal control.^16^ Similar benefits have also been shown for light-driven RAFT depolymerizations, whereby light has been demonstrated to accelerate the reaction and increase the monomer recovery yield.^17,18^ Electrochemical stimuli have also been studied, although lower conversions were achieved, most likely attributed to side reactions occurring in the presence of electricity.^19^ In addition, the use of chemical stimuli such as radical initiators has been proven highly beneficial to lower the reaction's temperature and boost the rate, *via* both ATRP and RAFT depolymerization protocols.^20,21^ However, radical initiators can be oxidizing agents and/or highly explosive chemicals, thereby posing challenges in both transportation and storage.^22–25^

In 2024, our group reported the acid-triggered polymerization of vinyl polymers in the absence of conventional radical initiators.^26^ By utilizing only sulfuric acid, low-dispersity polymers could be synthesized by RAFT polymerization with good control over molecular weight and excellent end-group fidelity, as exemplified for several chain extensions and the synthesis of multiblock copolymers.^27^ In comparison to conventional thermal RAFT polymerization, termination was suppressed and the acid was shown to possess a dual role in both initiating and accelerating the polymerization. Although the effect of acid on polymerizations has been documented for altering reactivity and tacticity,^28–31^ the impact of acidic additives in the chemical recycling of polymethacrylates remains underexplored, despite the potential to further improve depolymerization and reveal interesting mechanistic pathways.

Inspired by our earlier work on acid-triggered polymerization, we sought to replace radical initiators typically used in depolymerization with more practical additives. First, acetic acid was utilized as a means to accelerate the RAFT depolymerization rate and improve the final conversion at elevated concentrations. By carefully monitoring the UV loss *via* size exclusion chromatography (SEC), it was proposed that the addition of acid allows for better preservation of the end-group fidelity during depolymerization, thus elucidating the higher conversions recorded. Complementary density functional theory calculations suggest that acid could act to lower the activation barrier for the depropagation step, thus enabling a faster depolymerization. In addition to acetic acid, trifluoroacetic acid, citric acid, and a Lewis acid were also explored to expand the reaction scope, further highlighting the versatility and robustness of acid-enhanced RAFT depolymerization. We note that a complementary strategy focusing on the acid-assisted depolymerization of RAFT derived polmethacrylates has been concurrently reported by Sumerlin and co-workers.^32^

## Results and discussion

To begin our investigation into the effect of acid on RAFT depolymerization, we first synthesized a model polymer. We selected benzyl methacrylate (BzMA) as the monomer, as it is well-established in depolymerization studies and its high boiling point is particularly advantageous because it prevents monomer evaporation under the reaction conditions, allowing us to obtain highly accurate depolymerization conversions directly *via*^1^H NMR spectroscopy. We used 2-cyanopropyl dithiobenzoate (CPDB) as the chain transfer agent (CTA) and targeted a relatively low conversion (*i.e.*, 64%) to maximize initial end-group fidelity.^1^ After multiple precipitations in methanol to remove residual monomer, we isolated the purified PBzMA with a low final dispersity (*Đ* = 1.15, Fig. S1).

**Fig. 1:**
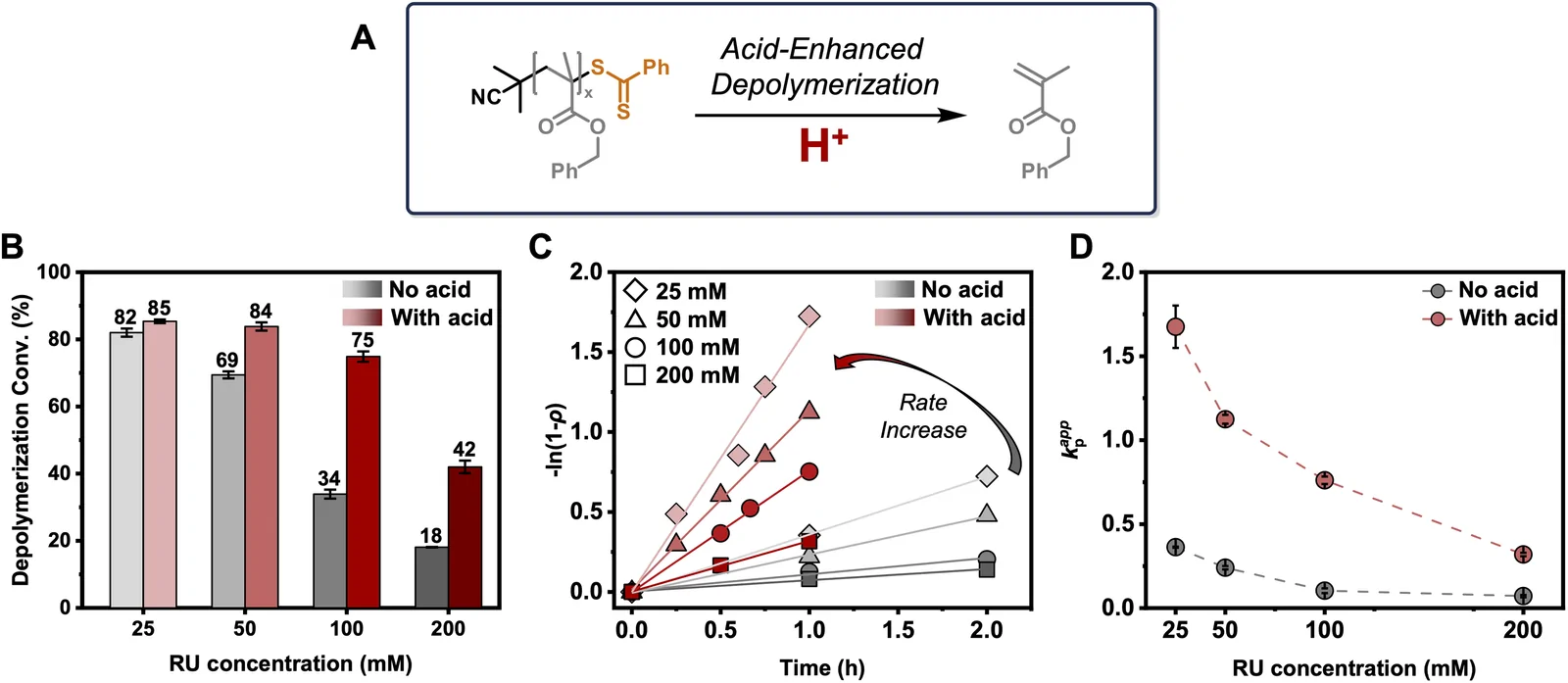
(A) Schematic representation of acid-enhanced depolymerization of dithiobenzoate-terminated poly(benzyl methacrylate). (B) Comparison of reaction conversion at varying repeating unit (RU) concentrations (25–200 mM) for the depolymerization of PBzMA in 1,4-dioxane at 120 °C over 5 hours with (red) and without (grey) the addition of acetic acid. (C) Pseudo-first order kinetic plots of depolymerization of PBzMA with and without the addition of acetic acid. (D) Apparent rate constants (*k*^app^_p_) as a function of RU concentration.

Initially, we decided to compare depolymerizations in the presence and absence of acid at a repeat unit concentration (RUC) of 50 mM, which provided a clear baseline for NMR analysis. We chose previously established reaction conditions, specifically utilizing dioxane at 120 °C (Fig. 1A),^7^ and selected acetic acid as our initial additive because it is benign and widely available and has previously been employed in acid-enhanced polymerization studies.^26^ In the absence of acid, the control reaction reached 69% depolymerization conversion after 5 hours, which is in line with previous literature values at the same repeat unit concentration^7^ (Fig. 1B and Table S1). We then repeated the reaction with varying amounts of acetic acid (Fig. S2). The addition of acid consistently increased monomer recovery, reaching a maximum of 84% conversion after 5 hours when using 0.65 M acetic acid, representing a 15% improvement. Analysis of the molecular weight evolution during depolymerization revealed no significant difference between reactions in the presence or absence of acid. While these initial results confirmed that acid has a positive effect on depolymerization, we hypothesized that conducting the reactions at higher concentrations might reveal an even greater contrast because conventional RAFT depolymerization typically suffers as the RUC increases.^13^ As shown in Fig. 1B, doubling the RUC to 100 mM caused the control reaction conversion to drop to 34% (without acid), whereas the acid-containing system achieved 75% conversion, more than doubling the reaction yield. We observed a similar trend at 200 mM, where the conversion without acid dropped to 18% but reached 42% with it, again more than doubling the original reaction efficiency. Interestingly, lowering the concentration to 25 mM yielded nearly identical results between the two setups, with 82% conversion for the control *versus* 85% with acid. This similarity is likely due to the fact that depolymerization at 25 mM without any additive already performs exceptionally well, effectively unzipping most of the chains containing the active RAFT end-group. Ultimately, these results demonstrate that the addition of acid offers a promising means of partially overcoming the concentration limitations typically associated with RAFT depolymerization, enabling high-efficiency monomer recovery at higher concentrations and significantly increasing the absolute yield of recycled material.

Encouraged by these final conversion trends, we next sought to investigate the kinetic profile of the depolymerization in greater detail. We repeated the reactions across the different repeat unit concentrations both with and without acid, sampling the mixtures at regular intervals to track the reaction progress over time. Fig. 1C shows the pseudo-first-order kinetic plots obtained for the control reactions and the acid-containing systems. As expected, lower RUCs yielded faster depolymerization rates across both series due to the reactions operating further from thermodynamic equilibrium. The more significant trend, however, is that the presence of acid accelerated the depolymerization rate across the entire concentration range. Remarkably, the depolymerization rate at 200 mM in the presence of acid was comparable to that of the 25 mM control reaction, demonstrating that acid can effectively compensate for the kinetic penalties associated with high concentration. To quantify this acceleration, we calculated and compared the apparent rate constants (*k*^app^_p_) for each system (Fig. 1D). At a standard concentration of 50 mM, the addition of acetic acid increased the depolymerization rate by a factor of 4.7, increasing *k*^app^_p_ from 0.24 h^−1^ to 1.12 h^−1^. This acceleratory effect became even more pronounced at 100 mM, where the acid-containing system exhibited a rate constant of 0.76 h^−1^ compared to just 0.10 h^−1^ for the control, representing a 7.6-fold increase in the reaction rate. Even at the highest concentration of 200 mM, the rate constant was more than quadrupled upon the addition of acid. Taken together, these kinetic profiles demonstrate a distinct acceleratory effect, but this alone did not fully explain the dramatic differences in final conversions. While an increased reaction rate implies faster monomer release, it was less clear why the presence of acid allowed the depolymerization to proceed to much higher overall yields before stalling, particularly at elevated concentrations. We speculated that this enhanced efficiency could be related to the intactness of the active chain ends over the course of the reaction. In other words, we hypothesized that the faster depolymerization rates encouraged end-group activation over end-group degradation.

**Fig. 2:**
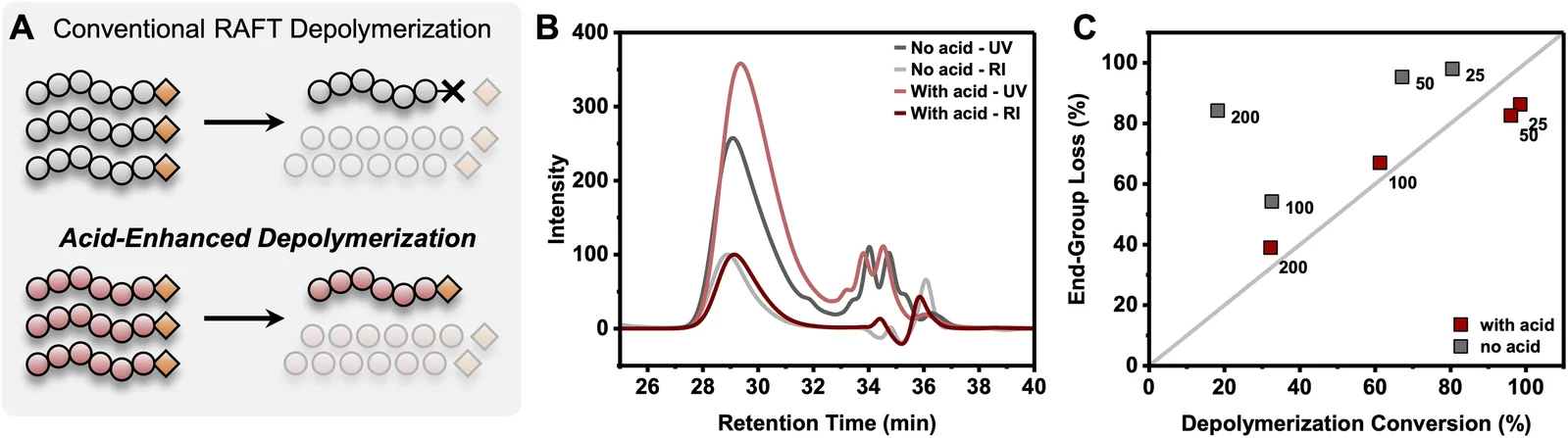
(A) Cartoon representation of the loss of end-group fidelity in conventional *vs.* acid-enhanced depolymerization. (B) UV and RI responses for depolymerization of PBzMA (100 mM) with and without acid at comparable conversions (∼30%). (C) End-group loss *vs.* depolymerization conversion (5 h) for a range of RU concentrations with and without acid.

To investigate this hypothesis, we considered the pathway depicted in Fig. 2A. If fewer polymer chains lose their thiocarbonylthio end groups to deleterious side reactions, such as irreversible termination or chain transfer, a larger fraction of the population remains capable of activation to form a productive, depropagating radical that unzips completely to monomer. We tested this by conducting parallel depolymerization experiments at an RUC of 100 mM, one in the presence of acetic acid and one without, stopping both reactions at an identical intermediate conversion of approximately 30% (Table S2). By sampling identical aliquots from each mixture, we were able to perform size exclusion chromatography (SEC) and directly compare the signals from the refractive index (RI) detector against the UV detector, which selectively monitors the UV-active RAFT end groups. As shown in Fig. 2B, both samples exhibit nearly identical peak areas in the RI traces, confirming that an equal mass of polymer had undergone depolymerization in each case. However, a stark contrast emerged in the UV signals, where the acid-treated polymer showed a significantly more intense signal compared to the control, indicating higher end-group fidelity, in line with our original hypothesis.

In an ideal end-group-initiated depolymerization, a chain is activated, loses its thiocarbonylthio group, and subsequently unzips completely to monomer. Under these conditions, a 1 : 1 correlation would exist between the percentage of end-group loss and the percentage of depolymerization conversion. To see how our system compared to this benchmark, we plotted end-group loss against depolymerization conversion across the entire range of examined concentrations from 25 mM to 200 mM (Fig. 2C). The resulting plot reveals that the data points for the acid-enhanced reactions lie significantly closer to the ideal than those of the control series. While the preservation of end-group fidelity explains why the acid-enhanced depolymerization achieves higher conversions, it also raises fundamental questions regarding the origin of this overall kinetic enhancement. In the context of acid-triggered RAFT polymerization, it has been proposed that acid plays a dual role by both facilitating a Flory-type autoinitiation to generate radicals and directly catalyzing the propagation step.^26^ We therefore first considered whether a similar radical-generation pathway could be active during depolymerization. Although there is no monomer present at the start of a depolymerization reaction, it was conceivable that the small amount of monomer generated as depropagation progresses could undergo acid-catalyzed autoinitiation, thereby introducing additional radicals into the system and accelerating the reaction.

To test this hypothesis, we conducted depolymerization kinetic studies at 50 mM RUC with the deliberate initial addition of small quantities of benzyl methacrylate monomer (2, 5, and 10 mM). These experiments revealed no meaningful difference in the reaction rate compared to the standard acid-enhanced depolymerization, indicating that the presence of monomer does not trigger additional radical generation (Fig. S3 and Table S3). This finding is further supported by two key facts. First, the absolute monomer concentration under these dilute depolymerization conditions is likely too low to favour classical autoinitiation pathways. Second, if autoinitiation by the liberated monomer were responsible for the acceleration, we would expect to see a distinct autocatalytic acceleration in the kinetic profiles over time as the monomer concentration builds, which was not observed in our kinetic data. Consequently, we could rule out acid-catalyzed autoinitiation as the source of the kinetic enhancement during depolymerization, leading us to consider the alternative hypothesis that acid directly alters the energetic barriers of the depropagation process itself.

**Fig. 3:**
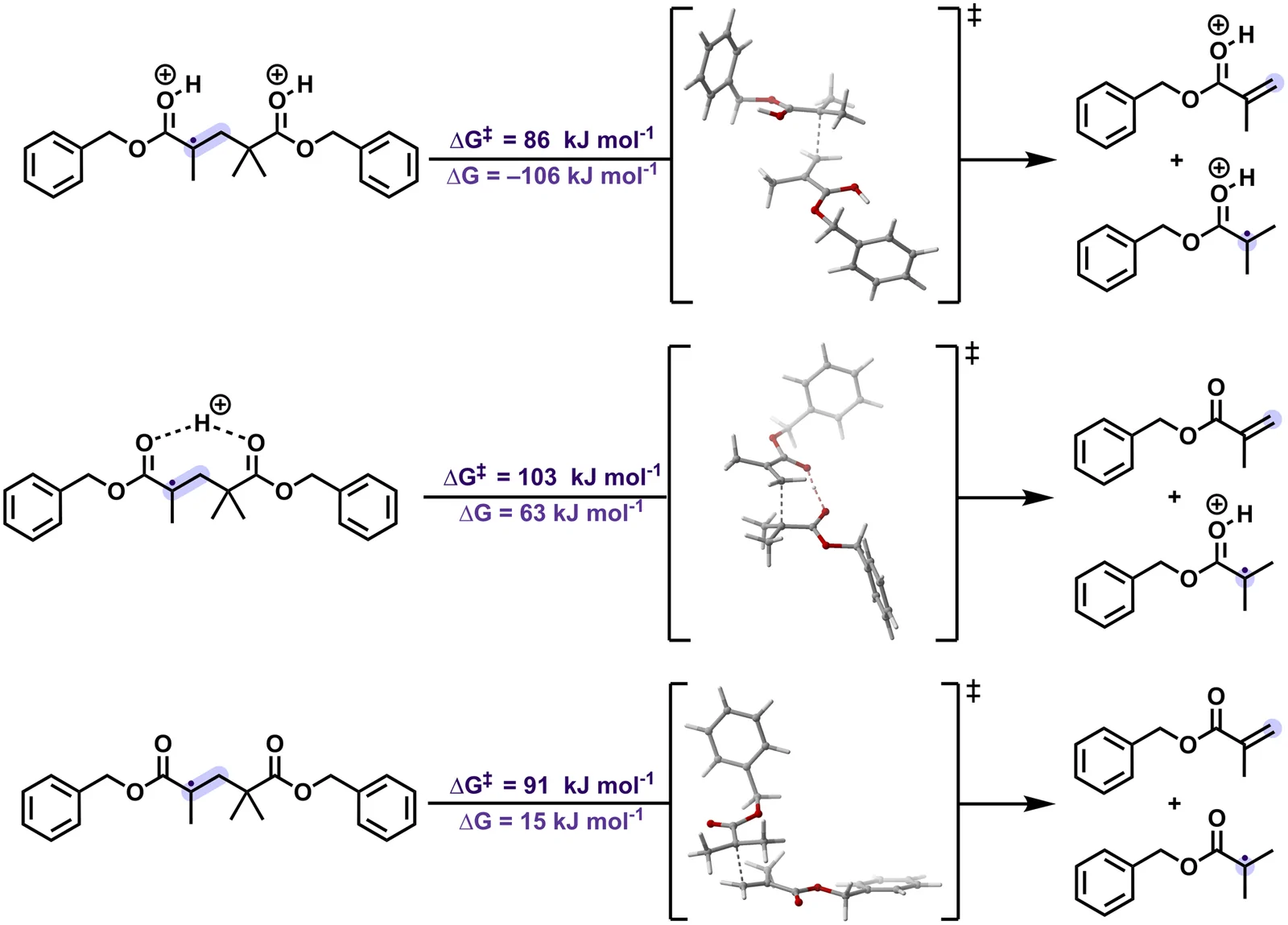
Gibbs free energy barriers and reaction energies (393.15 K, 1,4-dioxane) for depropagation of a PBzMA model system in the presence and absence of acid calculated at the SMD-wB97X-D/aug-cc-pVTZ//SMD-M062X/6-31G(d,p) level of theory.

To understand whether acid can catalyze the depropagation step, we used DFT to examine the effect of protonation on the kinetics and thermodynamics of depropagation for a model benzyl methacrylate dimer radical (Fig. 3, computational results, SI). We considered both singly and doubly protonated dimers and compared these with the corresponding neutral system. The singly protonated model was used to represent the effect of catalytic acid, whereas the doubly protonated model is more representative of stoichiometric acid conditions. In practice, the true protonation state depends not only on the acid concentration but also on the relevant p*K*_a_ values and the solvent properties. The models considered here are also highly simplified, as they neglect chain-length effects and microsolvation; nevertheless, they provide useful qualitative insights. For the singly protonated dimer, depropagation is both kinetically and thermodynamically less favourable than in the corresponding neutral system. This arises because, in the dimer radical, the proton is hydrogen-bonded to both the terminal and penultimate carbonyl groups, and depropagation disrupts this shared hydrogen bond. In contrast, for the doubly protonated dimer, depropagation is faster and considerably more exergonic than in the neutral system because the reaction relieves electrostatic repulsion. While these represent simplified limiting cases, the results suggest that acid has the potential to either inhibit or promote depropagation, with promotion becoming more likely at higher acid concentrations.

**Fig. 4:**
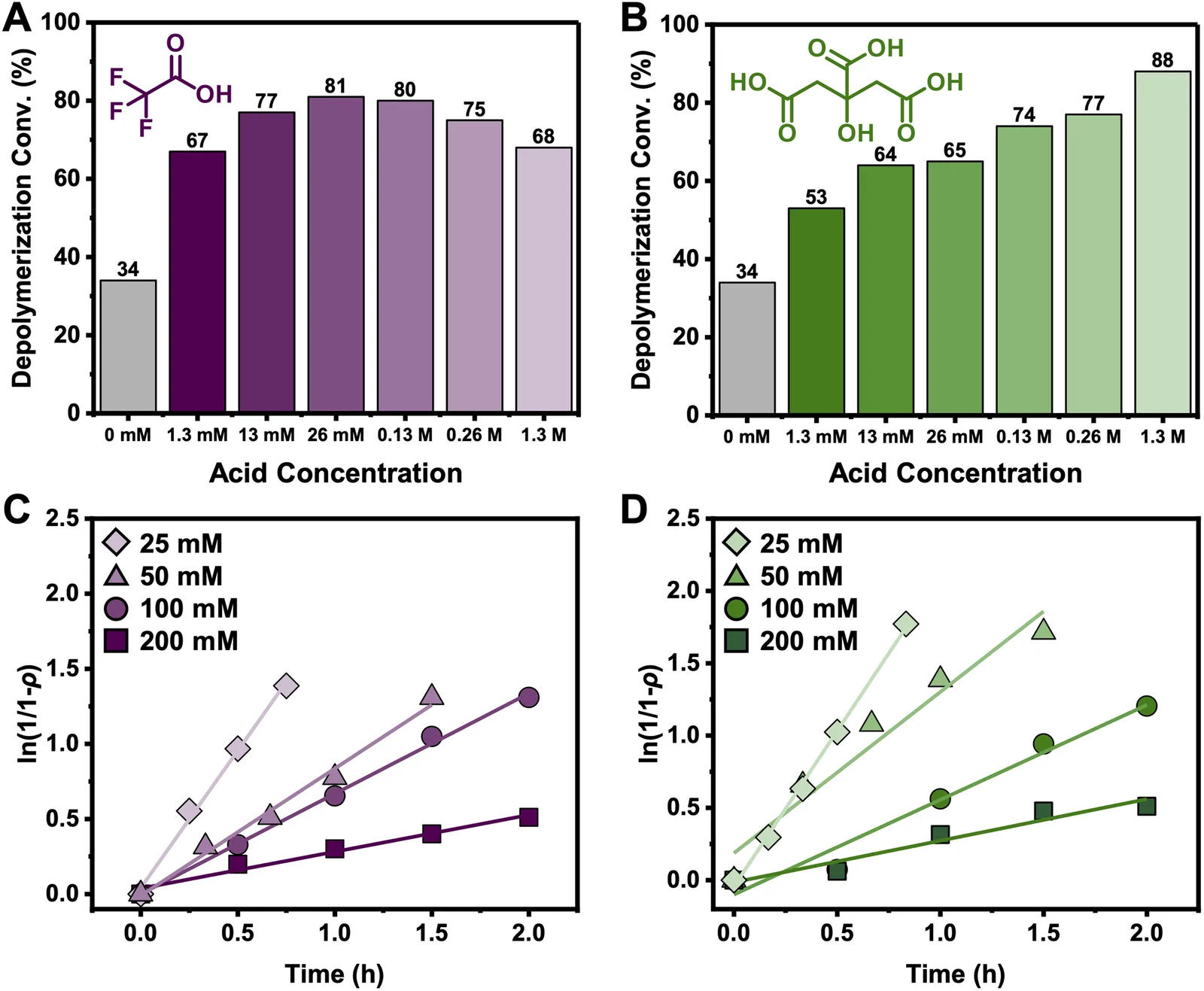
(A) Depolymerization conversion *vs.* trifluoroacetic acid concentration after 5 h. (B) Depolymerization conversion *vs.* citric acid concentration after 5 h. (C) Depolymerization kinetics for different repeat unit concentrations with optimized trifluoroacetic acid concentrations. (D) Depolymerization kinetics for different repeat unit concentrations with optimized citric acid concentrations.

Following the insights obtained from our DFT calculations, we were prompted to investigate how the strength and structure of the acidic additive affect the depolymerization process. We first selected trifluoroacetic acid (TFA), which is structurally analogous to acetic acid but possesses significantly greater acidity due to the electron-withdrawing fluorine atoms. As shown in Fig. 4A and Table S5, the addition of TFA successfully increased depolymerization conversion, reaching a maximum enhancement at a concentration of just 26 mM TFA after 5 h. This represents a substantially lower required loading compared to the 650 mM optimized for the weaker acetic acid system, a trend that aligns well with the computational prediction that stronger protonation environments favour depropagation. To explore a more sustainable alternative, we next evaluated citric acid, which is appealing because it is benign, safe, and a solid at room temperature, making it considerably easier to handle. Citric acid also successfully delivered enhanced depolymerization yields (Fig. 4B and Table S6), though its maximum effect was observed at higher concentrations, a consequence of it being a much weaker acid than TFA. We then conducted kinetic studies using the optimized amounts of each acid across a repeat unit concentration range of 25 mM to 200 mM (Fig. 4C and D and Tables S7 and S8). Mirroring our observations with acetic acid, both the TFA and citric acid systems exhibited marked kinetic acceleration compared to the acid-free control reactions. Ultimately, these results highlight the versatility of this approach, demonstrating that the structural benefits of acid-assisted depolymerization can be achieved across a range of acid strengths and chemical profiles.

Finally, to push the scope of this strategy even further, we explored whether the system could be extended to Lewis acids. We selected lithium bis(trifluoromethanesulfonimide), as triflamides have been documented to coordinate efficiently and accelerate radical reactions more effectively than traditional triflates.^29^ Pleasingly, at a repeat unit concentration of 100 mM and a fixed reaction time of 5 h, the addition of this Lewis acid led to an increase in depolymerization conversion, increasing the yield from approximately 40% in the control reaction to 75% (Fig. S4) even at remarkably low additive concentrations (1.3 mM). These results strongly imply that the coordination of a Lewis acid to the polymer backbone can influence the depropagation pathway just as effectively as Brønsted protonation, suggesting that further exploration of Lewis acid catalysis could offer a highly fruitful avenue for optimizing polymer recycling methodologies.

## Conclusions

In summary, we have shown that adding simple acidic additives is an effective strategy for enhancing RAFT-mediated depolymerization. By introducing acetic acid to poly(benzyl methacrylate), a higher percentage of monomer can be successfully regenerated. Kinetic analyses revealed a distinct rate acceleration across all repeating unit concentrations, including a 7.6-fold increase in the apparent rate constant at 100 mM. SEC analysis established that this faster depropagation leaves less time for deleterious side reactions to occur, bringing the system closer to an ideal unzipping mechanism. Furthermore, control experiments and density functional theory calculations allowed us to rule out acid-catalyzed autoinitiation and suggest that lowered depropagation activation barriers could be the cause of this acceleration. While these initial findings establish a clear foundation for acid-assisted depropagation, this work represents a preliminary communication of a highly tunable phenomenon. The successful expansion of this protocol to structurally diverse Brønsted acids like trifluoroacetic acid and sustainable citric acid, alongside a promising proof-of-concept exploration of Lewis acid catalysis, underscores the versatility of this approach. Future efforts will focus on clarifying the precise coordination mechanics of these additives and expanding the monomer scope. Ultimately, we anticipate that integrating Brønsted acid and Lewis acid catalysis into polymer deconstruction could offer a practical avenue for improving the efficiency and viability of chemical recycling technologies beyond RAFT-synthesized polymers.

## Author contributions

Z. K.: investigation. M.-N. A.: conceptualization, investigation, supervision, visualization, and writing – original draft. A. P. T.: investigation. A. A. K.: investigation, formal analysis, visualization, and writing – review & editing. N. P. T.: conceptualization and writing – review & editing. G. R. J.: conceptualization, investigation, visualization, and writing – original draft. M. L. C.: conceptualization, writing – original draft, and supervision. A. A.: conceptualization, writing – original draft, supervision, project administration, and funding acquisition. All authors have read and agreed to the published version of the manuscript.

## Conflicts of interest

The authors declare no conflicts of interest.

## Supplementary Material

SC-OLF-D6SC05503F-s001

## Data Availability

Experimental procedures, characterization data, and all other relevant data supporting the findings of this study are available within the article and its supplementary information (SI). Supplementary information is available. See DOI: https://doi.org/10.1039/d6sc05503f.
