# Supplementary material for: Acid-enhanced RAFT depolymerization of polymethacrylates
Source: Chem Sci. 2026 Sep 24;17(41):19815–20. doi: 10.1039/d6sc05503f (PMC13607952; doi:10.1039/d6sc05503f)
Supplement: Supplementary file 1 [file SC-017-D6SC05503F-s001.pdf]

Supplementary Information (SI) for Chemical Science.
This journal is © The Royal Society of Chemistry

Acid-Enhanced RAFT Depolymerization of Polymethacrylates

Zuzanna Kantor,[1,‡] Maria-Nefeli Antonopoulou,[1,‡,*] Athénaïs Pascal Touraine,[1] Asja A. Kroeger,[2] Nghia P. Truong,[1] Glen R. Jones,[1] Michelle L. Coote,[2] Athina Anastasaki[1,*]

[1] Laboratory for Sustainable Polymers, Department of Materials, ETH Zürich, Vladimir-Prelog-Weg 5, 8093 Zürich, Switzerland

[2] Institute for Nanoscale Science and Technology, College of Science and Engineering, Flinders University, South Australia, 5042 Australia

‡ These authors contributed equally

*Correspondence: m-nef@windowslive.com; athina.anastasaki@mat.ethz.ch

Contents

Materials 1

Instrumentation 1

Synthesis of PBzMA homopolymer. 2

Depolymerization of PBzMA in 1,4-Dioxane (RUC = 25 mM) 3

Depolymerization of PBzMA in 1,4-Dioxane (RUC = 50 mM) 4

Depolymerization of PBzMA in 1,4-Dioxane (RUC = 100 mM) 4

Depolymerization of PBzMA in 1,4-Dioxane (RUC = 200 mM) 4

Depolymerization of PBzMA in 1,4-Dioxane (RUC = 25 mM) with acetic acid 4

Depolymerization of PBzMA in 1,4-Dioxane (RUC = 50 mM) with acetic acid 5

Depolymerization of PBzMA in 1,4-Dioxane (RUC = 100 mM) with acetic acid 5

Depolymerization of PBzMA in 1,4-Dioxane (RUC = 200 mM) with acetic acid 5

Depolymerization of PBzMA in 1,4-Dioxane (RUC = 25 mM) with acetic acid in the presence of BzMA ... 6

Depolymerization of PBzMA in 1,4-Dioxane (RUC = 100 mM) with various trifluoroacetic acid concentrations 6

Depolymerization of PBzMA in 1,4-Dioxane (RUC = 100 mM) with various citric acid concentrations 6

Depolymerization of PBzMA in 1,4-Dioxane (RUC = 25 mM) with trifluoroacetic acid 7

Depolymerization of PBzMA in 1,4-Dioxane (RUC = 50 mM) with trifluoroacetic acid 7

Depolymerization of PBzMA in 1,4-Dioxane (RUC = 100 mM) with trifluoroacetic acid 8

Depolymerization of PBzMA in 1,4-Dioxane (RUC = 200 mM) with trifluoroacetic acid 8

Depolymerization of PBzMA in 1,4-Dioxane (RUC = 25 mM) with citric acid 8
Depolymerization of PBzMA in 1,4-Dioxane (RUC = 50 mM) with citric acid 9
Depolymerization of PBzMA in 1,4-Dioxane (RUC = 100 mM) with citric acid 9
Depolymerization of PBzMA in 1,4-Dioxane (RUC = 200 mM) with citric acid 9
Depolymerization of PBzMA in 1,4-Dioxane (RUC = 100 mM) with lithium bis(trifluoromethanesulfonimide) 10
Data corresponding to Figure 1 11
Data corresponding to Figure 2 12
Data corresponding to Figure 3 13
1. Computational Methods 13
2. Total Energies 15
3. Cartesian coordinates 15
Data corresponding to Figure 4 31
References 33

Materials

2-Cyano-2-propyl benzodithioate (CPDB), Benzyl methacrylate (BzMA), 1,4-Dioxane, glacial Acetic acid (AA), Citric acid (monohydrate) (CA), Trifluoroacetic acid (TfA), and Lithium bis(trifluoromethanesulfonimide) were purchased from Sigma Aldrich or Fischer Scientific. All chemicals were used as received except for benzyl methacrylate, which was passed twice through basic alumina to remove inhibitors.

Instrumentation

^{1}H NMR spectra were recorded on a DPX-300 spectrometer in acetone-d_6. Chemical shifts are given in ppm downfield from tetramethylsilane referenced to residual acetone-d_6 protons. Monomer conversions were determined via ^{1}H NMR spectroscopy by comparing the integrals of monomeric vinyl protons to polymer signals. SEC analysis of polymer samples was performed using a Shimadzu

modular system comprising of a CBM-20A system controller, an SIL-20A automatic injector, a 10.0 μm bead-size guard column (50 × 7.5 mm) followed by three KF-805L columns (300 × 8 mm, bead size: 10 μm, pore size maximum: 5000 Å), an SPD-20A ultraviolet detector, and an RID-20A differential refractive-index detector. The temperature of the columns was maintained at 40 °C using a CTO-20A oven. The eluent was *N,N*-dimethylacetamide (HPLC grade, with 0.03% w/v LiBr) and the flow rate was kept at 1 mL min^{-1} using an LC-20AD pump. A molecular weight calibration curve was produced using commercial narrow molecular weight distribution poly(methyl methacrylate) standards with molecular weights ranging from 5000 to 1.5×10^6.

SEC/UV Analysis

To compare end-group fidelity at equivalent depolymerization conversions, aliquots were taken from the acid-assisted and control reactions at identical conversions. Each aliquot was accurately weighed on an analytical balance, dried completely under a stream of air, and re-dissolved in a precisely measured volume of DMAc to yield solutions of identical concentration. All samples were filtered through a PTFE syringe filter prior to analysis. Exactly 100 μL of each sample was injected into the SEC instrument using identical integration limits across both runs, enabling direct quantitative comparison of the raw refractive index (RI) and ultraviolet (UV) signals without mathematical normalization.

For end-group retention measurements, aliquots were collected from the reaction mixture at t = 0 h and t = 5 h. Identical sample concentrations were maintained by accurately weighing each aliquot on an analytical balance, evaporating volatile components under air, and dissolving the remaining material in equal volumes of DMAc eluent. Samples were filtered through PTFE filters, and 100 μL

injection volumes were analyzed under identical SEC-UV instrumental settings. Because both initial (t = 0 h) and final (t = 5 h) samples were collected from the same acid-containing reaction medium at identical concentrations, direct comparison of raw peak areas was used to determine relative end-group preservation without requiring trace normalization or extinction coefficient adjustments.

Synthesis of PBzMA homopolymer.

In a 50 ml round-bottomed flask, 313.5 mg (1 eq.) of CPDB was dissolved in 11 ml of toluene. A stock solution of 70 mg AIBN in 3 ml toluene was prepared, and 1 ml was added to the reaction vessel. Subsequently, 12 ml of monomer was added to the reaction vessel, along with a magnetic stirring bar. The flask was then sealed with a rubber septum and purged with nitrogen for 20 minutes. Polymerization was conducted in an oil bath at 70 ᵒC and was stirred at 350 rpm. Reaction was stopped after 4 hours at 64% conversion. The polymer was then precipitated with cold methanol 3 times.

Depolymerization of PBzMA in 1,4-Dioxane (RUC = 25 mM)

In a 10mL test tube, 13.7 mg (2.0 μmol) of PBzMA was inserted and dissolved in 3mL of 1,4-Dioxane. Subsequently, the tube was sealed with a septum and purged with nitrogen for 20 minutes. The depolymerization was then conducted in an oil bath at 120 °C for 5 hours. Samples were taken periodically to extract the kinetic profile.

Depolymerization of PBzMA in 1,4-Dioxane (RUC = 50 mM)

In a 10mL test tube, 27.4 mg (3.9 μmol) of PBzMA was inserted and dissolved in 3mL of 1,4-Dioxane. Subsequently, the tube was sealed with a septum and purged with nitrogen for 20 minutes. The depolymerization was then conducted in an oil bath at 120 ˚C for 5 hours. Samples were taken periodically to extract the kinetic profile.

Depolymerization of PBzMA in 1,4-Dioxane (RUC = 100 mM)

In a 10mL test tube, 54.9 mg (7.8 μmol) of PBzMA was inserted and dissolved in 3mL of 1,4-Dioxane. Subsequently, the tube was sealed with a septum and purged with nitrogen for 20 minutes. The depolymerization was then conducted in an oil bath at 120 ˚C for 5 hours. Samples were taken periodically to extract the kinetic profile.

Depolymerization of PBzMA in 1,4-Dioxane (RUC = 200 mM)

In a 10mL test tube, 110 mg (15.7 μmol) of PBzMA was inserted and dissolved in 3mL of 1,4-Dioxane. Subsequently, the tube was sealed with a septum and purged with nitrogen for 20 minutes. The depolymerization was then conducted in an oil bath at 120 ˚C for 5 hours. Samples were taken periodically to extract the kinetic profile.

Depolymerization of PBzMA in 1,4-Dioxane (RUC = 25 mM) with acetic acid

In a 10mL test tube, 13.7 mg (2.0 μmol) of PBzMA was inserted and dissolved in 2.943 mL of 1,4-Dioxane. Subsequently, 56.5 μL (97.9 μmol) of glacial acetic acid was added, and then the tube was sealed with a septum and purged with nitrogen for 20 minutes. The depolymerization was then

conducted in an oil bath at 120 °C for 5 hours. Samples were taken periodically to extract the kinetic profile.

Depolymerization of PBzMA in 1,4-Dioxane (RUC = 50 mM) with acetic acid

In a 10mL test tube, 27.4 mg (3.9 μmol) of PBzMA was inserted and dissolved in 2.887 mL of 1,4-Dioxane. Subsequently, 113 μL (1.96 mmol) of glacial acetic acid was added, and then the tube was sealed with a septum and purged with nitrogen for 20 minutes. The depolymerization was then conducted in an oil bath at 120 °C for 5 hours. Samples were taken periodically to extract the kinetic profile.

Depolymerization of PBzMA in 1,4-Dioxane (RUC = 100 mM) with acetic acid

In a 10mL test tube, 54.9 mg (7.8 μmol) of PBzMA was inserted and dissolved in 2.774 mL of 1,4-Dioxane. Subsequently, 226 μL (3.92 mmol) of glacial acetic acid was added, and then the tube was sealed with a septum and purged with nitrogen for 20 minutes. The depolymerization was then conducted in an oil bath at 120 °C for 5 hours. Samples were taken periodically to extract the kinetic profile.

Depolymerization of PBzMA in 1,4-Dioxane (RUC = 200 mM) with acetic acid

In a 10mL test tube, 110 mg (15.7 μmol) of PBzMA was inserted and dissolved in 2.546 mL of 1,4-dioxane. Subsequently, 454 μL (7.86 mmol) of glacial acetic acid was added, and then the tube was sealed with a septum and purged with nitrogen for 20 minutes. The depolymerization was then conducted in an oil bath at 120 °C for 5 hours. Samples were taken periodically to extract the kinetic profile.

Depolymerization of PBzMA in 1,4-Dioxane (RUC = 25 mM) with acetic acid in the presence of BzMA

In a 10mL test tube, 13.7 mg (2.0 μmol) of PBzMA was inserted and dissolved in 2.943 mL of 1,4-dioxane. Subsequently, 56.5 μL (97.9 μmol) of glacial acetic acid was added, and then the tube was sealed with a septum and purged with nitrogen for 20 minutes. The depolymerization was then conducted in an oil bath at 120 ˚C for 5 hours. Samples were taken periodically to extract the kinetic profile.

Depolymerization of PBzMA in 1,4-Dioxane (RUC = 100 mM) with various trifluoroacetic acid concentrations

In a 10mL test tube, 54.9 (7.8 μmol) of PBzMA was inserted and dissolved in 3 mL of 1,4-dioxane. Subsequently, 0.3 μL (3.9 μmol, 1.3 mM) of trifluoroacetic acid was added, and then the tube was sealed with a septum and purged with nitrogen for 20 minutes. The depolymerization was then conducted in an oil bath at 120 ˚C for 5 hours. Similar procedures were followed for 13 mM, 26 mM, 0.13 M, 0.26 M, and 1.3 M, with a total reaction volume of 3 mL.

Depolymerization of PBzMA in 1,4-Dioxane (RUC = 100 mM) with various citric acid concentrations

In a 10mL test tube, 54.9 mg (7.8 μmol) of PBzMA was inserted. In a separate vial, 0.82 mg (3.9 μmol, 1.3 mM) of citric acid was inserted and then dissolved in 3 mL of 1,4-dioxane. The citric acid solution was then inserted into the tube containing the polymer. Subsequently, the tube was sealed

with a septum and purged with nitrogen for 20 minutes. The depolymerization was then conducted in an oil bath at 120 °C for 5 hours. Similar procedures were followed for 13 mM, 26 mM, 0.13 M, 0.26 M, and 1.3 M.

Depolymerization of PBzMA in 1,4-Dioxane (RUC = 25 mM) with trifluoroacetic acid

In a 10mL test tube, 13.7 mg (2.0 μmol) of PBzMA was inserted and dissolved in 3 mL of 1,4-dioxane. Subsequently, 1.5 μL (19.6 μmol, 6.5 mΜ) of trifluoroacetic acid was added, and then the tube was sealed with a septum and purged with nitrogen for 20 minutes. The depolymerization was then conducted in an oil bath at 120 °C for 5 hours. Samples were taken periodically to extract the kinetic profile.

Depolymerization of PBzMA in 1,4-Dioxane (RUC = 50 mM) with trifluoroacetic acid

In a 10mL test tube, 27.4 mg (3.9 μmol) of PBzMA was inserted and dissolved in 3 mL of 1,4-dioxane. Subsequently, 3.0 μL (39.1 μmol, 13 mΜ) of trifluoroacetic acid was added, and then the tube was sealed with a septum and purged with nitrogen for 20 minutes. The depolymerization was then conducted in an oil bath at 120 °C for 5 hours. Samples were taken periodically to extract the kinetic profile.

Depolymerization of PBzMA in 1,4-Dioxane (RUC = 100 mM) with trifluoroacetic acid

In a 10mL test tube, 54.9 mg (7.8 μmol) of PBzMA was inserted and dissolved in 2.994 mL of 1,4-dioxane. Subsequently, 6.0 μL (78.4 μmol, 26 mΜ) of trifluoroacetic acid was added, and then the tube was sealed with a septum and purged with nitrogen for 20 minutes. The depolymerization was then conducted in an oil bath at 120 ˚C for 5 hours. Samples were taken periodically to extract the kinetic profile.

Depolymerization of PBzMA in 1,4-Dioxane (RUC = 200 mM) with trifluoroacetic acid

In a 10mL test tube, 110 mg (15.7 μmol) of PBzMA was inserted and dissolved in 2.988 mL of 1,4-dioxane. Subsequently, 12.0 μL (157.1 μmol, 52 mΜ) of trifluoroacetic acid was added, and then the tube was sealed with a septum and purged with nitrogen for 20 minutes. The depolymerization was then conducted in an oil bath at 120 ˚C for 5 hours. Samples were taken periodically to extract the kinetic profile.

Depolymerization of PBzMA in 1,4-Dioxane (RUC = 25 mM) with citric acid

In a 10mL test tube, 13.7 mg (2.0 μmol) of PBzMA was inserted and dissolved in 3 mL of 1,4-dioxane. Subsequently, 206 mg (0.98 mmol, 0.33 M) of citric acid was added, and then the tube was sealed with a septum and purged with nitrogen for 20 minutes. The depolymerization was then conducted in an oil bath at 120 ˚C for 5 hours. Samples were taken periodically to extract the kinetic profile.

Depolymerization of PBzMA in 1,4-Dioxane (RUC = 50 mM) with citric acid

In a 10mL test tube, 27.4 mg (3.9 μmol) of PBzMA was inserted and dissolved in 3 mL of 1,4-dioxane. Subsequently, 411 mg (1.96 mmol, 0.65 Μ) of citric acid was added, and then the tube was sealed with a septum and purged with nitrogen for 20 minutes. The depolymerization was then conducted in an oil bath at 120 °C for 5 hours. Samples were taken periodically to extract the kinetic profile.

Depolymerization of PBzMA in 1,4-Dioxane (RUC = 100 mM) with citric acid

In a 10mL test tube, 54.9 mg (7.8 μmol) of PBzMA was inserted and dissolved in 3 mL of 1,4-dioxane. Subsequently, 824 mg (3.92 mmol, 1.3 M) of citric acid was added, and then the tube was sealed with a septum and purged with nitrogen for 20 minutes. The depolymerization was then conducted in an oil bath at 120 °C for 5 hours. Samples were taken periodically to extract the kinetic profile.

Depolymerization of PBzMA in 1,4-Dioxane (RUC = 200 mM) with citric acid

In a 10mL test tube, 110 mg (15.7 μmol) of PBzMA was inserted and dissolved in 3 mL of 1,4-dioxane. Subsequently, 1651 mg (7.86 mmol, 2.6 M) of citric acid was added, and then the tube was sealed with a septum and purged with nitrogen for 20 minutes. The depolymerization was then conducted in an oil bath at 120 °C for 5 hours. Samples were taken periodically to extract the kinetic profile.

Depolymerization of PBzMA in 1,4-Dioxane (RUC = 100 mM) with lithium bis(trifluoromethanesulfonimide)

In a 10mL test tube, 54.9 mg (7.8 μmol) of PBzMA was inserted. In a separate vial, 1.13 mg (3.9 μmol, 1.3 mM) of lithium bis(trifluoromethanesulfonimide) was inserted and then dissolved in 3 mL of 1,4-dioxane. The lithium bis(trifluoromethanesulfonimide) solution was then inserted into the tube containing the polymer. Subsequently, the tube was sealed with a septum and purged with nitrogen for 20 minutes. The depolymerization was then conducted in an oil bath at 120 °C for 5 hours. Similar procedures were followed for 13 mM and 26 mM.

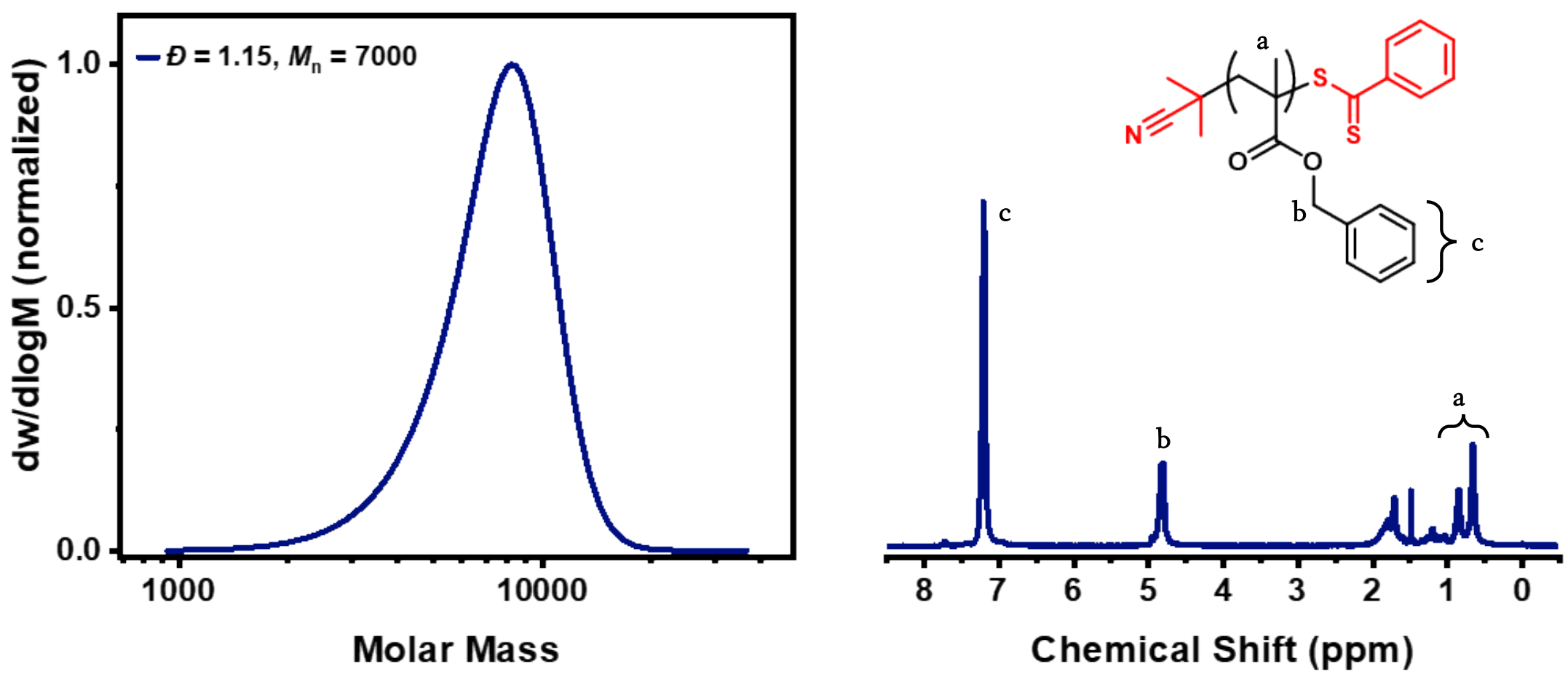

Figure S 1. Molecular weight distribution and ^{1}H NMR of PBzMA after purification.

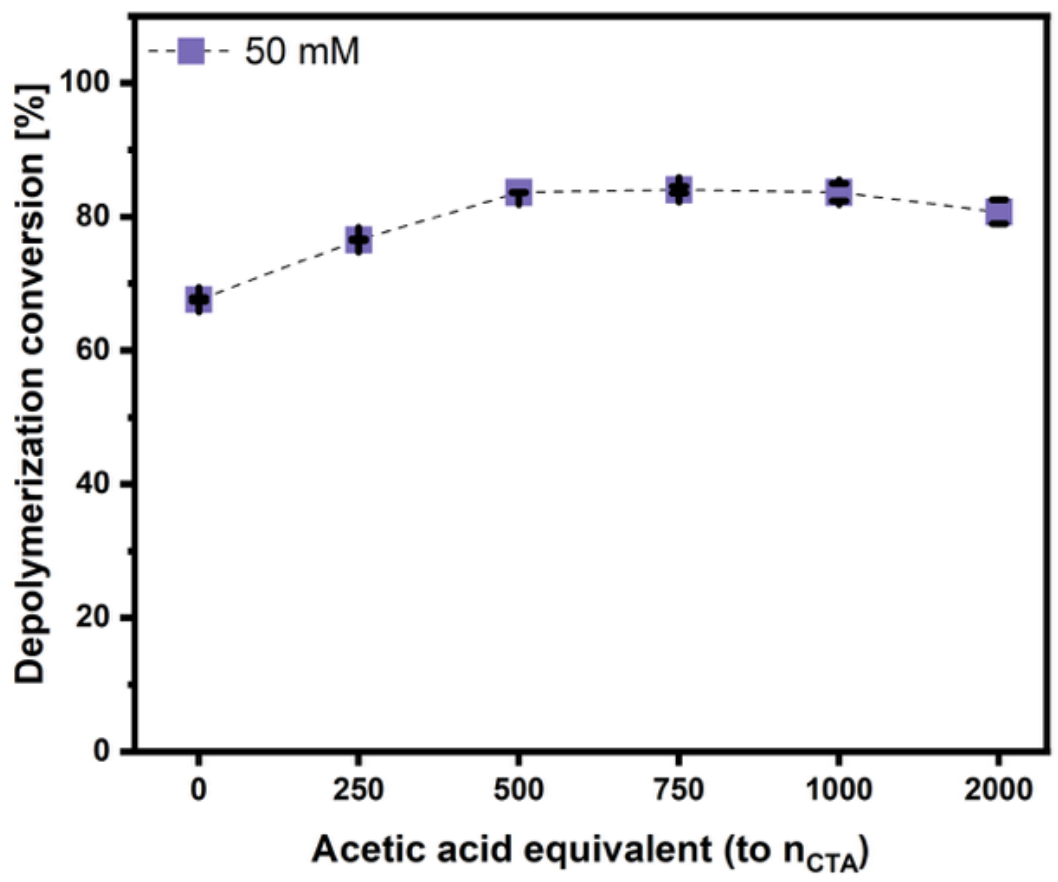

Figure S 2: Depolymerization of PBzMA with differing amounts of acetic acid.

Data corresponding to Figure 1

Table S 1. Data corresponding to Figure 1.

Entry	PBzMA (mg)	Dioxane (mL)	[RU] (mM)	Acetic acid (μL)	Temperature (℃)	Time (h)	k_p^{app} (h^{-1})
1	13.7	3.000	25	-	120	5	0.36
2	13.7	2.943	25	56.5	120	5	**1.68**
3	27.4	3.000	50	-	120	5	0.24
4	27.4	2.887	50	113	120	5	**1.12**
5	54.9	3.000	100	-	120	5	0.10
6	54.9	2.774	100	226	120	5	**0.76**
7	110.0	3.000	200	-	120	5	0.07
8	110.0	2.546	200	454	120	5	**0.32**

Data corresponding to Figure 2

Table S 2. Data corresponding to Figure 2.

Entry	PBzMA (mg)	Dioxane (mL)	[RU] (mM)	Acetic acid (μL)	Temperature (℃)	Time (h)	Depol. (%)[a]
1	54.9	3.000	100	-	120	3.0	29
2	54.9	2.774	100	226	120	0.5	30

[a] Conversion was calculated by ^{1}H NMR.

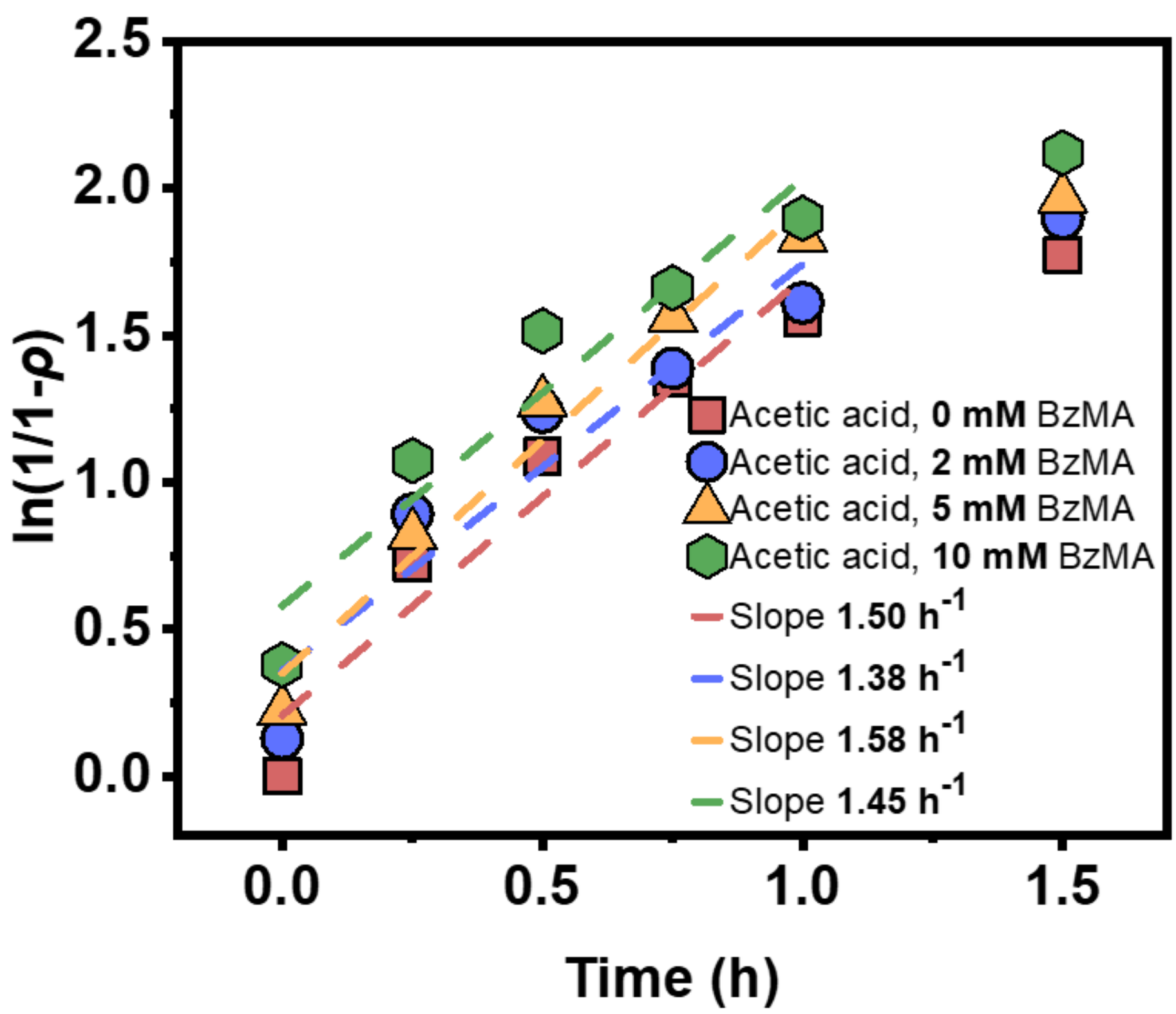

Figure S 3. Kinetic profiles of the depolymerization of PBzMA at [RU] = 25 mM in the presence of acid, with 0 mM, 2 mM, 5 mM, and 10 mM of BzMA.

Table S 3. Data corresponding to Figure S 2.

Entry	PBzMA (mg)	Dioxane (mL)	[RU] (mM)	Acetic acid (μL)	BzMA (μL)	Temperature (℃)	Time (h)	k_p^{app} (h^{-1})
1	13.7	2.943	25	56.5	-	120	5	**1.50**
2	13.7	2.943	25	56.5	1.0	120	5	**1.38**

**3**	13.7	2.943	25	56.5	2.5	120	5	**1.58**
**4**	13.7	2.943	25	56.5	5.1	120	5	**1.45**

Data corresponding to Figure 3

1. Computational Methods

Geometry optimizations of all equilibrium and transition structures along the neutral and acid catalyzed model depropagation processes were performed at the SMD[1]-(1,4-dioxane)-M062X[2]/6-31G(d,p)[3] level of theory. To confirm equilibrium structures as minima with no imaginary frequencies and transition structures as first order saddle points characterized by a single imaginary frequency reflecting the motion along the depropagation reaction coordinate of interest, frequency calculations were carried out on all optimized structures at the SMD[1]-(1,4-dioxane)-M062X[2]/6-31G(d,p)[3] level of theory. To further confirm the connectivities between optimized transition structures and depropagation reactants and products, intrinsic reaction coordinates (IRCs)[4] were calculated at the same level of theory that was used for geometry optimizations and frequency calculations. To ensure accurate reaction energies and barriers, extensive conformational searches were carried out on all equilibrium and transition structures. Hereby, the full conformational space was searched with typical resolutions of 120° around sp^3-sp^3 bonds and 180° around sp^2-sp^3 bonds for all structures along the depropagation of the neutral system. To assess the effect of acid on depropagation while keeping the conformational space to be searched tractable, the resulting lowest energy conformers of the neutral system were protonated and smaller conformational searches were carried out around the protonation and reaction sites. The electronic energies of all resulting

geometries were then refined at the SMD[1]-(1,4-dioxane)-wB97X-D[5]/aug-cc-pVTZ[6] level of theory. The "direct method" by Truhlar and co-workers,[7] which applies ideal gas partition functions evaluated under the harmonic oscillator / rigid rotor approximation directly to solution-phase geometries and frequencies was used to obtain refined Gibbs free energies. A change of state correction to account for the change of state from 1 atm to a standard state of 1 M[8] was further applied. The Gaussian16[9] program package was used in all geometry optimizations, frequency, IRC, and single point energy calculations. 3D representations were generated using CYLview.[10]

2. Total Energies

Table S 4. Electronic energies (E_e), zero point energies (E_o), enthalpy (H), Gibbs free energies (G), thermal corrections (TC) for T = 393.15 K, and entropy (S) in Hartree obtained at the SMD-(1,4-dioxane)-wB97X-D/aug-cc-pVTZ//SMD-(1,4-dioxane)-M062X/6-31G(d,p) level of theory for all structures along the neutral and acid catalyzed depropagation reactions of a dimer pBzMA model.

T=393.15K (120 °C)	E_e	E_o	$H_{393.15}$	$G_{393.15}$	$G_{393.15}$ (1M)	TC	S	TS
**Free depolymerization products**								
Neutral BzMA	-576.85847	-576.65134	-576.62993	-576.71194	-576.70762	0.227297	547.665	0.082009
Charged BzMA	-577.24456	-577.02451	-577.00315	-577.08109	-577.07677	0.240161	520.517	0.077944
Neutral BzMA radical	-577.44302	-577.22636	-577.20377	-577.28948	-577.28516	0.238007	572.391	0.085711
Charged BzMA radical	-577.83476	-577.60461	-577.58189	-577.66596	-577.66164	0.251629	561.458	0.084074
**BzMA dimer radicals**								
Neutral BzMA dimer radical	-1154.3389	-1153.91	-1153.8669	-1154.0027	-1153.9984	0.47082	907.022	0.13582
Charged BzMA dimer radical (1 H^+)	-1154.753	-1154.3114	-1154.269	-1154.3976	-1154.3933	0.482744	859.248	0.128666
Charged BzMA dimer radical (2 H^+)	-1155.0794	-1154.6221	-1154.5804	-1154.7025	-1154.6982	0.497787	815.315	0.122088
**Depropagation transition structures**								
Neutral depropagation TS	-1154.2995	-1153.874	-1153.8304	-1153.9679	-1153.9635	0.467868	918.092	0.137478
Charged depropagation TS (1 H^+)	-1154.7089	-1154.2714	-1154.2289	-1154.3586	-1154.3543	0.47872	866.076	0.129689
Charged depropagation TS (2 H^+)	-1155.0461	-1154.5892	-1154.5476	-1154.6699	-1154.6656	0.497176	816.46	0.122259
**Product Complexes**								
Neutral Product Complex	-1154.3097	-1153.8844	-1153.8392	-1153.981	-1153.9766	0.469242	946.685	0.141759
Charged Product Complex (1 H^+)	-1154.7243	-1154.2833	-1154.2406	-1154.3686	-1154.3642	0.482496	854.605	0.127971
Charged Product Complex (2 H^+)	-1155.0598	-1154.5969	-1154.556	-1154.6771	-1154.6727	0.502525	808.394	0.121051

3. Cartesian coordinates

Cartesian coordinates (in Å) for all structures optimized at the SMD-(1,4-dioxane)-M062X/6-31G(d,p) level of theory along with their charges, multiplicities and imaginary frequencies, where applicable. The number of imaginary frequencies is zero for all equilibrium structures and one for all transition structures.

=== Free depolymerization products ===

Neutral BzMA

0 1
C -4.373203 0.329127 0.000237
H -4.711216 -0.701768 0.000133
H -5.122029 1.115157 0.000435

C	-3.071376	0.614005	0.000116
C	-2.124041	-0.546255	-0.000152
O	-2.444712	-1.713068	-0.000483
C	-2.503985	2.003809	0.000361
H	-1.873642	2.170192	0.878598
H	-1.872532	2.170101	-0.877078
H	-3.307144	2.743300	-0.000153
C	0.133535	-1.193947	-0.000205
H	-0.022624	-1.818439	0.884210
H	-0.022702	-1.818095	-0.884842
C	1.493750	-0.557680	-0.000103
C	2.125462	-0.244212	1.204065
C	2.125207	-0.243338	-1.204158
C	3.372739	0.372879	1.205964
H	1.635249	-0.487632	2.143118
C	3.372492	0.373761	-1.205860
H	1.634822	-0.486053	-2.143309
C	3.997559	0.682044	0.000095
H	3.858591	0.609815	2.147184
H	3.858148	0.611387	-2.147008
H	4.972130	1.159990	0.000152
O	-0.844774	-0.138070	0.000033

Charged BzMA

1 1

C	-3.304574	1.635973	0.367549
H	-2.693923	2.489743	0.093988
H	-4.300529	1.834854	0.749692
C	-2.870312	0.373359	0.245755
C	-1.527804	0.176648	-0.286190
O	-0.830096	1.193912	-0.662554
C	-3.654171	-0.854384	0.611159
H	-3.769821	-1.520515	-0.247594
H	-3.156407	-1.418230	1.404788
H	-4.644377	-0.562584	0.961726
C	0.173188	-1.373316	-1.024769
H	0.209092	-2.455497	-0.908859
H	0.066166	-1.120379	-2.083819
C	1.339523	-0.676492	-0.382634
C	2.171681	0.139427	-1.156618

C	1.582002	-0.826725	0.987038
C	3.244765	0.802419	-0.560953
H	1.994282	0.238071	-2.225269
C	2.648467	-0.158960	1.574458
H	0.940585	-1.469832	1.583739
C	3.478291	0.655444	0.801721
H	3.893165	1.427851	-1.164653
H	2.839255	-0.275569	2.635905
H	4.310416	1.172910	1.267082
O	-1.106410	-1.030897	-0.374524
H	0.117802	0.994700	-0.829193

Neutral BzMA radical

0 2

C	-2.800312	0.297917	0.110633
C	-3.622274	0.599028	1.311583
H	-3.178194	0.172402	2.210905
H	-3.726718	1.683314	1.445638
H	-4.638955	0.202689	1.191800
C	-1.588586	-0.481620	0.276561
O	-1.156187	-0.892303	1.339370
C	-3.244350	0.743115	-1.240791
H	-2.417528	1.166810	-1.818355
H	-3.627956	-0.103339	-1.826065
H	-4.042028	1.485467	-1.162650
C	0.277909	-1.410421	-0.814128
H	0.463844	-1.789995	-1.822884
H	0.183162	-2.257823	-0.129702
C	1.408150	-0.508723	-0.374500
C	2.575372	-1.074927	0.139448
C	1.326229	0.875966	-0.511668
C	3.648553	-0.269376	0.505667
H	2.640348	-2.153572	0.259558
C	2.397358	1.683794	-0.137992
H	0.417895	1.321109	-0.906781
C	3.561890	1.114252	0.368434
H	4.549896	-0.721368	0.907852
H	2.320959	2.761610	-0.243667
H	4.396031	1.744567	0.659814
O	-0.966362	-0.723434	-0.909987

Charged BzMA radical

1 2

C	2.967399	0.625450	0.050900
C	4.418276	0.323920	0.086404
H	4.922697	1.028859	0.752889
H	4.638737	-0.698900	0.389798
H	4.839580	0.481012	-0.916665
C	2.022217	-0.432533	-0.032354
O	2.465151	-1.660257	-0.115031
H	1.770407	-2.338028	-0.165961
C	2.492093	2.028540	0.102494
H	2.457839	2.355325	1.151684
H	3.207162	2.679259	-0.407071
H	1.496609	2.159752	-0.321065
C	-0.259817	-1.199931	-0.117667
H	-0.105307	-1.882896	0.725539
H	-0.109442	-1.713403	-1.073477
C	-1.601944	-0.548028	-0.052259
C	-2.243403	-0.391401	1.176862
C	-2.201993	-0.076779	-1.220908
C	-3.484505	0.233328	1.236059
H	-1.773338	-0.762506	2.083717
C	-3.442257	0.548172	-1.159748
H	-1.700969	-0.205730	-2.176730
C	-4.082268	0.701915	0.068374
H	-3.986933	0.350797	2.190022
H	-3.912716	0.908799	-2.068120
H	-5.053051	1.184300	0.113799
O	0.760591	-0.142565	-0.023381

=== BzMA dimer radicals ===

Neutral BzMA dimer radical

0 2

C	1.610136	2.251353	0.273736
C	2.637574	3.384628	0.288848

H	2.961765	3.633301	-0.723679
H	2.199759	4.274721	0.750052
H	3.520563	3.098405	0.868128
C	2.216238	1.063937	-0.467553
C	0.377871	2.716099	-0.566690
H	0.746318	3.033525	-1.549722
H	-0.047445	3.583898	-0.053810
C	-0.676590	1.677109	-0.746611
C	-1.816389	1.701696	0.154427
O	-1.985032	2.510859	1.051731
O	2.943420	1.165682	-1.429780
C	1.190035	1.890504	1.698508
H	0.446378	1.092095	1.716875
H	2.055203	1.559062	2.280582
H	0.758685	2.769399	2.185523
C	-0.589861	0.648620	-1.823961
H	-0.685221	-0.364150	-1.417293
H	-1.408088	0.773030	-2.545093
H	0.354180	0.726545	-2.368878
O	-2.700990	0.710594	-0.107218
O	1.799328	-0.115917	0.017470
C	-3.847183	0.682480	0.738632
H	-4.372270	1.642194	0.665066
H	-3.529315	0.570525	1.781045
C	2.286950	-1.289285	-0.662952
H	1.558220	-2.064750	-0.418855
H	2.281526	-1.107871	-1.740183
C	-4.745164	-0.454387	0.325378
C	-4.592013	-1.121464	-0.888867
C	-5.783712	-0.827199	1.182084
C	-5.467423	-2.147649	-1.239833
H	-3.784405	-0.838711	-1.555204
C	-6.659520	-1.847300	0.829221
H	-5.904680	-0.313677	2.133017
C	-6.502689	-2.512481	-0.385429
H	-5.337531	-2.662791	-2.186656
H	-7.461927	-2.127381	1.504479
H	-7.182832	-3.311963	-0.661342
C	3.661900	-1.669653	-0.179538
C	4.797789	-1.341761	-0.920793
C	3.808038	-2.337762	1.038253
C	6.063549	-1.685848	-0.453764

H	4.681967	-0.804667	-1.857092
C	5.072195	-2.677161	1.508802
H	2.923911	-2.592697	1.617260
C	6.202119	-2.352692	0.760716
H	6.942364	-1.429990	-1.037193
H	5.176686	-3.198233	2.455238
H	7.189290	-2.620261	1.124438

Charged BzMA dimer radical (1 H+)

1 2

C	1.596887	2.024544	-0.210559
C	2.801683	2.478684	-1.056078
H	3.118756	1.695864	-1.752322
H	2.527108	3.365670	-1.632427
H	3.646951	2.731128	-0.413962
C	2.032668	0.771370	0.530995
C	0.429458	1.693099	-1.180488
H	0.702686	0.810372	-1.768957
H	0.367833	2.533565	-1.880098
C	-0.940532	1.532859	-0.596170
O	1.506941	-0.339990	0.430568
C	-1.470188	0.216377	-0.363659
O	-0.754403	-0.840176	-0.301233
H	0.266536	-0.620745	-0.099259
C	-1.850445	2.696978	-0.428110
H	-2.597170	2.704231	-1.234555
H	-2.408771	2.644488	0.510772
H	-1.296574	3.634524	-0.471676
C	1.225753	3.113219	0.805749
H	0.397714	2.807829	1.453407
H	2.084803	3.350732	1.436292
H	0.932713	4.023400	0.274690
O	-2.756214	0.118317	-0.240071
O	3.079946	0.960748	1.289737
C	-3.336020	-1.209461	0.017582
H	-2.919852	-1.566805	0.962483
H	-3.012974	-1.867176	-0.792071
C	3.717609	-0.218819	1.869160
H	4.374080	0.201860	2.630929
H	2.953541	-0.839545	2.339559

C	4.479826	-0.962943	0.808504
C	4.113246	-2.256176	0.439608
C	5.554527	-0.339291	0.168615
C	4.822121	-2.927579	-0.553599
H	3.271522	-2.736492	0.930711
C	6.254495	-1.005036	-0.831125
H	5.843122	0.667571	0.460137
C	5.889181	-2.301555	-1.191191
H	4.537780	-3.937040	-0.832065
H	7.089724	-0.518137	-1.323944
H	6.439307	-2.823474	-1.967561
C	-4.821760	-1.044643	0.066647
C	-5.563453	-1.061896	-1.115972
C	-5.468946	-0.850258	1.287213
C	-6.941862	-0.883788	-1.078439
H	-5.058101	-1.217797	-2.065556
C	-6.848875	-0.675428	1.325586
H	-4.892070	-0.842890	2.208171
C	-7.584082	-0.690800	0.143082
H	-7.515830	-0.900623	-1.998909
H	-7.350261	-0.531323	2.276787
H	-8.660492	-0.557122	0.173310

Charged BzMA dimer radical (2 H+)

2 2

C	-0.879344	1.560174	0.165419
C	-0.406674	1.126504	1.556829
H	-0.104241	0.076328	1.591550
H	0.443840	1.748506	1.844599
H	-1.199763	1.275246	2.293843
C	-2.083153	0.744036	-0.240029
C	0.247168	1.357130	-0.914286
H	1.010950	2.101580	-0.673368
H	-0.157818	1.599527	-1.902850
C	0.845230	-0.009861	-0.961576
C	2.044555	-0.280041	-0.226374
O	2.582580	0.676573	0.466485
O	-2.205258	-0.435784	0.249876
C	-1.270805	3.044664	0.161764
H	-1.528985	3.397099	-0.838474

H	-2.120390	3.219220	0.827852
H	-0.424122	3.627330	0.529248
C	0.256761	-1.112336	-1.763407
H	0.977774	-1.467945	-2.510383
H	0.019009	-1.979644	-1.135917
H	-0.638887	-0.783712	-2.291916
O	2.519077	-1.478011	-0.289028
O	-2.910968	1.265842	-1.053139
C	3.668451	-1.925223	0.526186
H	3.836984	-2.933069	0.149312
H	3.314169	-1.975597	1.559675
C	-4.101534	0.563603	-1.615703
H	-4.630368	1.381951	-2.101110
H	-3.718236	-0.130795	-2.367805
C	4.866449	-1.031975	0.369976
C	5.425036	-0.816278	-0.895548
C	5.415225	-0.402245	1.493644
C	6.522328	0.024068	-1.030538
H	5.008105	-1.313759	-1.767240
C	6.518584	0.439396	1.349938
H	4.993167	-0.582603	2.479808
C	7.067582	0.652620	0.090292
H	6.960989	0.183966	-2.009706
H	6.946922	0.919697	2.223012
H	7.926117	1.305974	-0.021077
C	-4.908539	-0.126396	-0.557328
C	-5.015158	-1.523698	-0.569242
C	-5.523448	0.616459	0.456158
C	-5.735200	-2.173597	0.432789
H	-4.553024	-2.095487	-1.372108
C	-6.238753	-0.038288	1.450666
H	-5.451841	1.701061	0.453803
C	-6.340841	-1.430622	1.440895
H	-5.826243	-3.253704	0.417150
H	-6.723189	0.534794	2.233762
H	-6.902856	-1.934533	2.219925
H	3.490916	0.470230	0.788886
H	-3.075940	-0.869188	0.062842

=== Depropagation transition structures ===

Neutral depropagation TS

0 2

C	-1.868670	2.297111	-0.629275
C	-2.694581	3.541426	-0.575349
H	-3.123175	3.692898	0.416619
H	-2.105066	4.418232	-0.859508
H	-3.526821	3.466998	-1.289139
C	-2.297041	1.193867	0.238149
C	-0.215085	2.946759	0.815513
H	-0.984934	3.107706	1.564689
H	0.009982	3.759622	0.131892
C	0.674132	1.931389	0.954020
C	1.802113	1.893306	-0.005008
O	2.009964	2.701997	-0.885801
O	-3.053311	1.315637	1.186188
C	-1.091819	2.009708	-1.872565
H	-0.354189	1.221053	-1.718554
H	-1.770811	1.678677	-2.670939
H	-0.578149	2.908571	-2.224187
C	0.534813	0.841062	1.973746
H	0.570531	-0.146432	1.504061
H	1.350404	0.877637	2.704860
H	-0.411735	0.944515	2.510972
O	2.604622	0.832014	0.209846
O	-1.711555	0.022146	-0.090351
C	3.723133	0.722724	-0.668954
H	4.333298	1.629437	-0.584142
H	3.368072	0.669279	-1.703615
C	-2.083693	-1.111348	0.711780
H	-1.261045	-1.818286	0.584100
H	-2.145827	-0.809742	1.759805
C	4.521093	-0.502601	-0.307518
C	4.368325	-1.154354	0.915341
C	5.466420	-0.976541	-1.219971
C	5.151966	-2.265863	1.219203
H	3.631694	-0.793501	1.624963
C	6.251721	-2.082146	-0.913847
H	5.585589	-0.475211	-2.177548
C	6.095344	-2.731763	0.309057
H	5.022724	-2.768501	2.172771

H	6.982011	-2.440971	-1.632327
H	6.704068	-3.597857	0.548397
C	-3.383095	-1.713761	0.242194
C	-4.582913	-1.425540	0.894205
C	-3.395696	-2.554105	-0.873200
C	-5.778260	-1.977087	0.440648
H	-4.572111	-0.756272	1.748765
C	-4.589670	-3.102239	-1.330502
H	-2.462024	-2.779503	-1.382636
C	-5.783265	-2.815147	-0.671399
H	-6.706923	-1.750260	0.954914
H	-4.589399	-3.756584	-2.196628
H	-6.715558	-3.244992	-1.024255

Number of imaginary frequencies = 1 (-452.5i cm^{-1})

Charged depropagation TS (1 H+)

1 2

C	-1.129217	2.116266	-0.229398
C	-2.139051	2.901586	0.548667
H	-2.556991	2.333426	1.381115
H	-1.691623	3.825777	0.922818
H	-2.971350	3.180423	-0.111076
C	-1.450731	0.710618	-0.551742
C	0.379414	1.572472	1.323034
H	0.128541	0.515802	1.317376
H	-0.085135	2.165005	2.105161
C	1.599722	1.986042	0.829821
O	-0.620017	-0.103598	-0.993807
C	2.311340	1.104596	-0.079432
O	1.782316	0.156927	-0.741602
H	0.699021	0.103323	-0.818470
C	2.220546	3.308332	1.155759
H	3.142787	3.165515	1.729126
H	2.489396	3.864811	0.252390
H	1.535033	3.909647	1.754709
C	-0.352665	2.855467	-1.279179
H	0.392850	2.232445	-1.777351
H	-1.051496	3.209524	-2.048025
H	0.135266	3.735880	-0.852534
O	3.577581	1.338674	-0.217346

O	-2.686831	0.363384	-0.313547
C	4.369611	0.479645	-1.106058
H	5.265804	1.075769	-1.275681
H	3.820914	0.357273	-2.041220
C	-3.055581	-1.032243	-0.552105
H	-2.901419	-1.243225	-1.612558
H	-2.377268	-1.655844	0.035795
C	-4.486329	-1.194019	-0.141280
C	-5.496730	-1.229420	-1.101227
C	-4.816365	-1.288853	1.212450
C	-6.827223	-1.361582	-0.712618
H	-5.241357	-1.157699	-2.154956
C	-6.144609	-1.414947	1.601893
H	-4.028402	-1.267328	1.961069
C	-7.150967	-1.452390	0.638084
H	-7.609521	-1.394316	-1.463660
H	-6.396109	-1.490809	2.654698
H	-8.187695	-1.556722	0.941395
C	4.674446	-0.835381	-0.449552
C	5.528285	-0.876690	0.655732
C	4.112715	-2.015347	-0.935775
C	5.812087	-2.089749	1.272047
H	5.972809	0.042573	1.028408
C	4.407115	-3.231550	-0.325319
H	3.445927	-1.979300	-1.792865
C	5.252931	-3.268069	0.779626
H	6.476977	-2.119131	2.128969
H	3.974724	-4.148779	-0.711048
H	5.481453	-4.215856	1.256256

Number of imaginary frequencies = 1 (-573.6i cm^{-1})

Charged depropagation TS (2 H+)

2 2

C	1.152689	2.022637	-0.444892
C	0.393335	1.746791	-1.699557
H	0.101692	0.700843	-1.807299
H	-0.482122	2.396041	-1.761976
H	1.041550	1.997006	-2.551342
C	2.077515	1.022435	0.018641
C	-0.409181	1.538343	1.158174

H	-1.007838	2.324960	0.707982
H	0.307815	1.832647	1.919847
C	-0.786995	0.227994	1.096675
C	-1.932169	-0.122201	0.274047
O	-2.537677	0.798626	-0.401521
O	1.975719	-0.179681	-0.460013
C	1.390566	3.439178	-0.043400
H	1.672744	3.546800	1.005208
H	2.215232	3.840800	-0.647094
H	0.507842	4.044802	-0.253730
C	-0.103322	-0.875407	1.847385
H	-0.789938	-1.346606	2.557001
H	0.252988	-1.660479	1.173617
H	0.738824	-0.470811	2.413800
O	-2.287334	-1.353142	0.268883
O	2.941148	1.363745	0.913045
C	-3.379547	-1.873209	-0.586257
H	-3.436001	-2.915123	-0.274731
H	-3.017596	-1.815493	-1.616215
C	3.845394	0.388643	1.554215
H	4.461407	1.027475	2.186193
H	3.220238	-0.251540	2.184277
C	-4.667678	-1.127496	-0.390925
C	-5.267240	-1.065049	0.872369
C	-5.266565	-0.492501	-1.485379
C	-6.457093	-0.367154	1.033079
H	-4.807648	-1.569290	1.718473
C	-6.463603	0.203237	-1.316477
H	-4.813687	-0.570737	-2.471898
C	-7.053500	0.266278	-0.058916
H	-6.930184	-0.326416	2.008034
H	-6.934311	0.685146	-2.166431
H	-7.987327	0.801816	0.073861
C	4.656386	-0.387301	0.553908
C	4.580816	-1.785061	0.521785
C	5.487161	0.288822	-0.347348
C	5.338696	-2.502490	-0.403633
H	3.951876	-2.310607	1.237868
C	6.233901	-0.431449	-1.269912
H	5.555321	1.372675	-0.315252
C	6.160522	-1.825270	-1.297604
H	5.293523	-3.586279	-0.415277

H 6.884971 0.090665 -1.962765
H 6.753893 -2.381411 -2.015310
H -3.415498 0.523229 -0.756218
H 2.746861 -0.755131 -0.249645
Number of imaginary frequencies = 1 (-427.8*i* cm^{-1})

=== Product Complexes ===

Neutral Product Complex

0 2
C 2.039125 2.211391 0.936498
C 2.673442 3.547619 0.775650
H 3.204577 3.624738 -0.173365
H 1.921462 4.344037 0.838923
H 3.386957 3.731351 1.590076
C 2.305059 1.195648 -0.065778
C -0.036640 3.134585 -1.124312
H 0.826600 3.231639 -1.776364
H -0.293479 3.969640 -0.479638
C -0.772050 2.021579 -1.111601
C -1.925741 1.985272 -0.161821
O -2.190371 2.837844 0.656856
O 2.987670 1.369072 -1.065192
C 1.100633 1.986371 2.069558
H 0.920255 0.927360 2.253006
H 1.486554 2.449211 2.984481
H 0.132743 2.464855 1.856923
C -0.509200 0.825484 -1.979331
H -0.483968 -0.090176 -1.382885
H -1.298626 0.704304 -2.728538
H 0.447172 0.940569 -2.495189
O -2.658544 0.870498 -0.314743
O 1.706557 0.010334 0.211280
C -3.783121 0.749860 0.557196
H -4.450644 1.604026 0.395956
H -3.440122 0.799124 1.595995
C 2.060045 -1.081049 -0.651813
H 1.249505 -1.802837 -0.528005
H 2.084640 -0.736260 -1.687729

C	-4.491131	-0.550591	0.281148
C	-4.219009	-1.324717	-0.845402
C	-5.473208	-0.973772	1.180081
C	-4.921634	-2.507768	-1.067761
H	-3.454902	-1.002770	-1.544480
C	-6.176326	-2.151315	0.955043
H	-5.686220	-0.375316	2.062736
C	-5.900996	-2.923756	-0.171950
H	-4.700169	-3.105187	-1.946753
H	-6.936393	-2.469535	1.661604
H	-6.446206	-3.845533	-0.347913
C	3.380968	-1.688808	-0.252931
C	4.553954	-1.361876	-0.934959
C	3.442445	-2.570668	0.828266
C	5.770366	-1.916909	-0.545966
H	4.505678	-0.660396	-1.762010
C	4.657289	-3.122778	1.221015
H	2.530018	-2.825374	1.361866
C	5.823662	-2.797144	0.531581
H	6.677721	-1.659640	-1.083466
H	4.694583	-3.809897	2.060571
H	6.772179	-3.229875	0.834122

Charged Product Complex (1 H+)

1 2

C	1.407766	2.201646	-0.837464
C	0.189067	2.757177	-1.478880
H	-0.485201	2.000437	-1.879569
H	-0.358101	3.391134	-0.765637
H	0.495891	3.416356	-2.299139
C	1.574496	0.791066	-0.642265
C	-0.683695	1.319812	1.876219
H	-0.218697	1.812636	2.724563
H	-0.289483	0.351213	1.585725
C	-1.742754	1.861342	1.269117
C	-2.349216	1.103431	0.141558
O	-1.736159	0.310821	-0.587728
O	0.658785	-0.097078	-0.730262
H	-0.372939	0.186252	-0.666969
C	-2.409117	3.139938	1.686134

H	-1.831438	3.628530	2.471800
H	-2.516418	3.830199	0.844017
H	-3.414540	2.938643	2.065789
C	2.537037	3.106439	-0.496734
H	3.043961	2.805608	0.422701
H	3.289261	3.076801	-1.297380
H	2.184552	4.135147	-0.406290
O	-3.619753	1.351368	-0.029693
O	2.786573	0.398165	-0.379528
C	-4.338272	0.586551	-1.047589
H	-5.223198	1.193877	-1.236806
H	-3.720762	0.539519	-1.945645
C	3.031266	-1.034238	-0.178241
H	2.415620	-1.356010	0.665161
H	2.702971	-1.554562	-1.080948
C	-4.692727	-0.778476	-0.530503
C	-3.992052	-1.906354	-0.958300
C	-5.725099	-0.917714	0.400419
C	-4.324284	-3.164745	-0.464055
H	-3.186959	-1.793770	-1.678507
C	-6.051589	-2.173327	0.899856
H	-6.274768	-0.039616	0.729536
C	-5.351292	-3.297788	0.466312
H	-3.781995	-4.040631	-0.804941
H	-6.855922	-2.277381	1.620740
H	-5.610317	-4.278662	0.851706
C	4.495038	-1.205263	0.078562
C	5.363806	-1.471188	-0.980199
C	5.000198	-1.075196	1.373321
C	6.728457	-1.607843	-0.746390
H	4.968665	-1.576391	-1.987065
C	6.364381	-1.209642	1.607462
H	4.321054	-0.872175	2.197627
C	7.228003	-1.476212	0.547014
H	7.400877	-1.821199	-1.570654
H	6.753914	-1.112094	2.615353
H	8.291888	-1.586563	0.730395

Charged Product Complex (2 H+)

2 2

C	1.583649	2.349960	-0.634239
C	0.735656	2.156539	-1.837712
H	0.409433	1.124738	-1.969509
H	-0.133227	2.819391	-1.814609
H	1.320568	2.436270	-2.726234
C	2.298124	1.235815	-0.104221
C	-0.775447	1.741694	1.320302
H	-1.425458	2.468916	0.844664
H	-0.025711	2.116434	2.011598
C	-0.912869	0.422216	1.122052
C	-1.996924	-0.010992	0.240397
O	-2.638380	0.866463	-0.452024
O	2.051470	0.052386	-0.593882
C	1.791516	3.704286	-0.065664
H	2.233583	3.689293	0.930295
H	2.464281	4.268626	-0.726856
H	0.845238	4.252271	-0.049374
C	-0.104426	-0.650538	1.794711
H	-0.722719	-1.219652	2.494511
H	0.303423	-1.359764	1.068999
H	0.712884	-0.195161	2.358681
O	-2.242955	-1.263683	0.205229
O	3.154770	1.445616	0.847456
C	-3.320000	-1.860340	-0.622199
H	-3.304238	-2.898559	-0.294099
H	-2.985240	-1.794174	-1.660261
C	3.830152	0.342118	1.548308
H	4.504227	0.869968	2.221776
H	3.067382	-0.181939	2.133892
C	-4.642657	-1.188077	-0.393304
C	-5.197732	-1.140027	0.890941
C	-5.310320	-0.591469	-1.469176
C	-6.410631	-0.494366	1.092274
H	-4.687941	-1.617882	1.723420
C	-6.530021	0.052088	-1.259288
H	-4.890195	-0.656552	-2.470886
C	-7.074699	0.102393	0.019072
H	-6.846772	-0.464280	2.085026
H	-7.051885	0.506899	-2.094006
H	-8.023765	0.601930	0.181816
C	4.555360	-0.580453	0.607649
C	4.235666	-1.943175	0.588647

C	5.528274	-0.078111	-0.263648
C	4.889959	-2.800284	-0.296113
H	3.496195	-2.332867	1.286287
C	6.171011	-0.935772	-1.147017
H	5.781671	0.978298	-0.244642
C	5.852900	-2.294886	-1.163054
H	4.651895	-3.858449	-0.300117
H	6.925143	-0.548793	-1.824150
H	6.361385	-2.960175	-1.852559
H	-3.485031	0.536705	-0.834392
H	2.726392	-0.619468	-0.345799

Data corresponding to Figure 4

Table S 5. Data corresponding to Figure 4A.

Entry	PBzMA (mg)	Dioxane (mL)	[RU] (mM)	Trifluoroacetic acid (μL)	[Acid]	Temperature (℃)	Time (h)
1	54.9	3.000	100	-	-	120	5
2	54.9	3.000	100	0.3	1.3 mM	120	5
3	54.9	3.000	100	3.0	13 mM	120	5
4	54.9	3.000	100	6.0	26 mM	120	5
5	54.9	2.970	100	30.0	0.13 M	120	5
6	54.9	2.940	100	60.1	0.26 M	120	5
7	54.9	2.700	100	300.3	1.3 M	120	5

Table S 6. Data corresponding to Figure 4B.

Entry	PBzMA (mg)	Dioxane (mL)	[RU] (mM)	Citric acid (mg)	[Acid]	Temperature (℃)	Time (h)
1	54.9	3.000	100	-	-	120	5
2	54.9	3.000	100	0.82	1.3 mM	120	5
3	54.9	3.000	100	8.2	13 mM	120	5
4	54.9	3.000	100	16.5	26 mM	120	5
5	54.9	3.000	100	82.4	0.13 M	120	5

6	54.9	3.000	100	164.8	0.26 M	120	5
7	54.9	3.000	100	824.0	1.3 M	120	5

Table S 7. Data corresponding to Figure 4C.

Entry	PBzMA (mg)	Dioxane (mL)	[RU] (mM)	Trifluoroacetic acid (μL)	[Acid]	Temperature (℃)	k_p^{app} (h^{-1})
1	13.7	3.000	25	1.5	6.5 mM	120	**1.83**
2	27.4	3.000	50	3.0	13.0 mM	120	**0.85**
3	54.9	2.994	100	6.0	26.0 mM	120	**0.67**
4	110.0	2.988	200	12.0	52.0 mM	120	**0.24**

Table S 8. Data corresponding to Figure 4D.

Entry	PBzMA (mg)	Dioxane (mL)	[RU] (mM)	Citric acid (mg)	[Acid]	Temperature (℃)	k_p^{app} (h^{-1})
1	13.7	3.000	25	206	0.33 M	120	**2.15**
2	27.4	3.000	50	411	0.65 M	120	**1.12**
3	54.9	3.000	100	824	1.3 M	120	**0.76**
4	110.0	3.000	200	1651	2.6 M	120	**0.29**

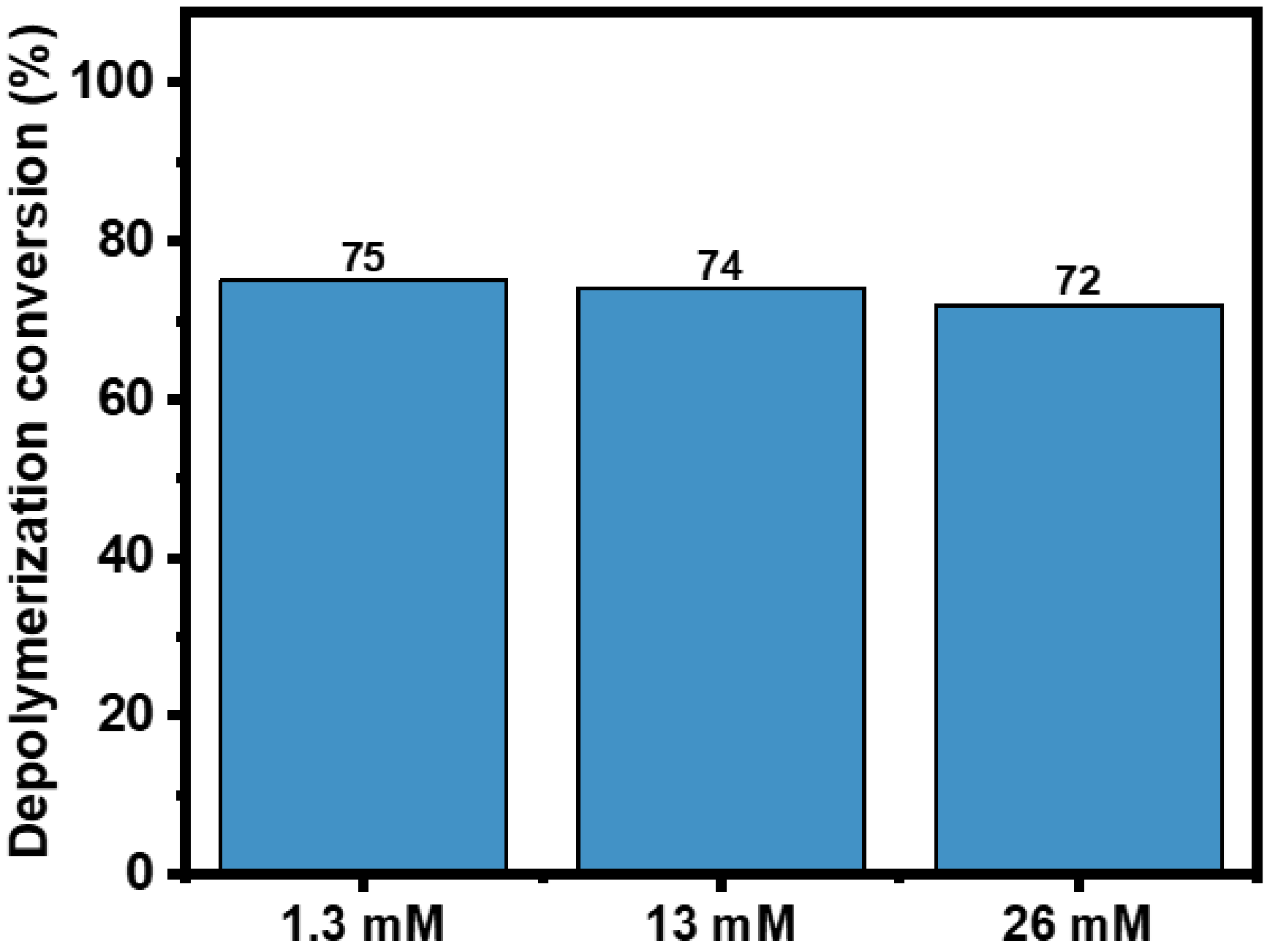

Figure S 4. Depolymerization conversion with different concentrations of lithium bis(trifluoromethanesulfonimide).

References

[1] A. V. Marenich, C. J. Cramer and D. G. Truhlar, *The Journal of Physical Chemistry B* **2009**, *113*, 6378-6396.

[2] Y. Zhao and D. G. Truhlar, *Theoretical Chemistry Accounts* **2008**, *120*, 215-241.

[3] a) W. J. Hehre, R. Ditchfield and J. A. Pople, *The Journal of Chemical Physics* **1972**, *56*, 2257-2261; b) P. C. Hariharan and J. A. Pople, *Theoretica chimica acta* **1973**, *28*, 213-222; c) M. M. Francl, W. J. Pietro, W. J. Hehre, J. S. Binkley, M. S. Gordon, D. J. DeFrees and J. A. Pople, *The Journal of Chemical Physics* **1982**, *77*, 3654-3665; d) M. S. Gordon, J. S. Binkley, J. A. Pople, W. J. Pietro and W. J. Hehre, *Journal of the American Chemical Society* **1982**, *104*, 2797-2803.

[4] C. Gonzalez and H. B. Schlegel, *The Journal of Chemical Physics* **1989**, *90*, 2154-2161.

[5] J.-D. Chai and M. Head-Gordon, *Physical Chemistry Chemical Physics* **2008**, *10*, 6615-6620.

[6] a) T. H. Dunning, Jr., *The Journal of Chemical Physics* **1989**, *90*, 1007-1023; b) R. A. Kendall, T. H. Dunning, Jr. and R. J. Harrison, *The Journal of Chemical Physics* **1992**, *96*, 6796-6806.

[7] R. F. Ribeiro, A. V. Marenich, C. J. Cramer and D. G. Truhlar, *The Journal of Physical Chemistry B* **2011**, *115*, 14556-14562.

[8] J. Ho, A. Klamt and M. L. Coote, *The Journal of Physical Chemistry A* **2010**, *114*, 13442-13444.

[9] M. J. Frisch, G. W. Trucks, H. B. Schlegel, G. E. Scuseria, M. A. Robb, J. R. Cheeseman, G. Scalmani, V. Barone, G. A. Petersson, H. Nakatsuji, X. Li, M. Caricato, A. V. Marenich, J. Bloino, B. G. Janesko, R. Gomperts, B. Mennucci, H. P. Hratchian, J. V. Ortiz, A. F. Izmaylov, J. L. Sonnenberg, Williams, F. Ding, F. Lipparini, F. Egidi, J. Goings, B. Peng, A. Petrone, T. Henderson, D. Ranasinghe, V. G. Zakrzewski, J. Gao, N. Rega, G. Zheng, W. Liang, M. Hada, M. Ehara, K. Toyota, R. Fukuda, J. Hasegawa, M. Ishida, T.

Nakajima, Y. Honda, O. Kitao, H. Nakai, T. Vreven, K. Throssell, J. A. Montgomery Jr., J. E. Peralta, F. Ogliaro, M. J. Bearpark, J. J. Heyd, E. N. Brothers, K. N. Kudin, V. N. Staroverov, T. A. Keith, R. Kobayashi, J. Normand, K. Raghavachari, A. P. Rendell, J. C. Burant, S. S. Iyengar, J. Tomasi, M. Cossi, J. M. Millam, M. Klene, C. Adamo, R. Cammi, J. W. Ochterski, R. L. Martin, K. Morokuma, O. Farkas, J. B. Foresman and D. J. Fox in *Gaussian 16 Rev. C.01*, *Vol.* Wallingford, CT, **2016**.
[10] CYLview20; C. Y. Legault, Université de Sherbrooke, 2020, http:// www.cylview.org.
